# *meso*-α,α-5,15-Bis(*o*-nicotinamido­phen­yl)-10,20-diphen­ylporphyrin *n*-hexane monosolvate

**DOI:** 10.1107/S2414314623010854

**Published:** 2023-12-22

**Authors:** Xiaotao Sun, Jianfeng Li

**Affiliations:** a College of Materials Science and Optoelectronic Technology, CAS Center for Excellence in Topological Quantum Computation & Center of Materials Science and Optoelectronics Engineering, University of Chinese Academy of Sciences, Yanqi Lake, Huairou District, Beijing 101408, People’s Republic of China; Benemérita Universidad Autónoma de Puebla, México

**Keywords:** crystal structure, porphyrins, solvate, hydrogen bonds, disorder

## Abstract

The structure of the title solvated porphyrin, C_56_H_38_N_8_O_2_·C_6_H_14_, is reported. Two porphyrin mol­ecules, one ordered and one disordered *n*-hexane solvate mol­ecules are present in its asymmetric unit. The porphyrin macrocycle shows a characteristic saddle-shaped distortion, and the maximum deviation from the mean plane for non-hydrogen atoms is 0.48 Å. N—H⋯N, N—H⋯O, and C—H⋯O hydrogen bonds, as well as π*–*π inter­actions, are observed in the crystal structure.

## Abstract 2

In the title porphyrin solvate, the macrocycle shows a characteristic saddle-shaped distortion.

## Graphical abstract

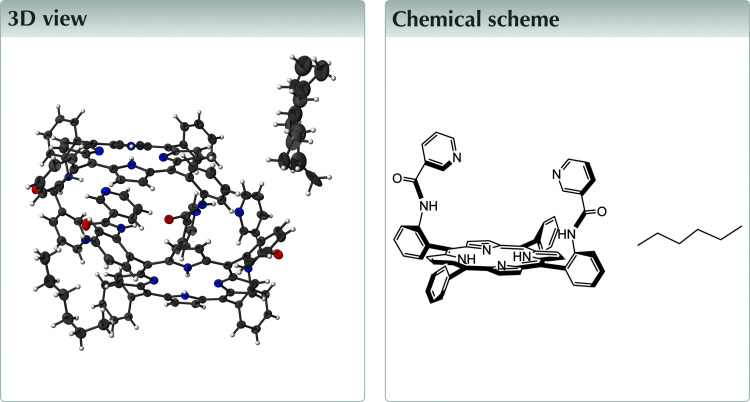

## Structure description

The characterization of a large class of porphyrins and their metallated derivatives has generated considerable inter­est because of their applications in catalysis and the preparation of new functional materials. For example, they are useful in photodynamic therapy (Ethirajan *et al.*, 2011 ▸; Bonnett, 1995 ▸; Peters *et al.*, 2018 ▸), as catalysts in nature (Shultz *et al.*, 2009 ▸; Li & Zamble, 2009 ▸), for important materials for dye-sensitized solar cells (Urbani *et al.*, 2014 ▸), or as responsive contrast agents in functional magnetic resonance imaging (Venkataramani *et al.*, 2011 ▸; Dommaschk *et al.*, 2015 ▸). Additionally, they are present throughout the biosphere and perform a wide range of bioinorganic functions (Averill, 1996 ▸). The presence or absence of a metal ion at the porphyrin core can greatly affect its physical properties, such as catalytic activity and crystal packing. Therefore, the design and synthesis of structurally diverse mol­ecules are essential. Herein, we report the structural properties of a new solvated porphyrin compound, C_56_H_38_N_8_O_2_·C_6_H_14_.

**Figure 1:**
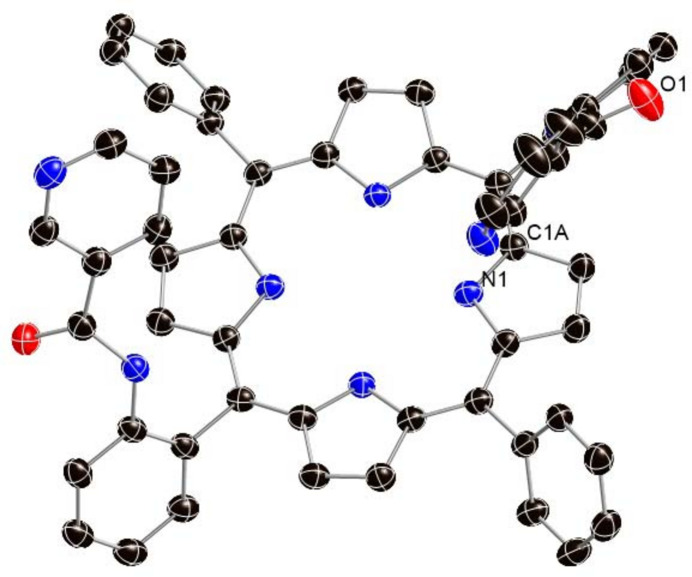
Top view of the porphyrin complex of the title compound with ellipsoids drawn at the 50% probability level. Hydrogen atoms, a disordered *n*-hexane solvate mol­ecule, an ordered *n*-hexane solvate mol­ecule and one porphyrin mol­ecule are omitted for clarity.

**Figure 3:**
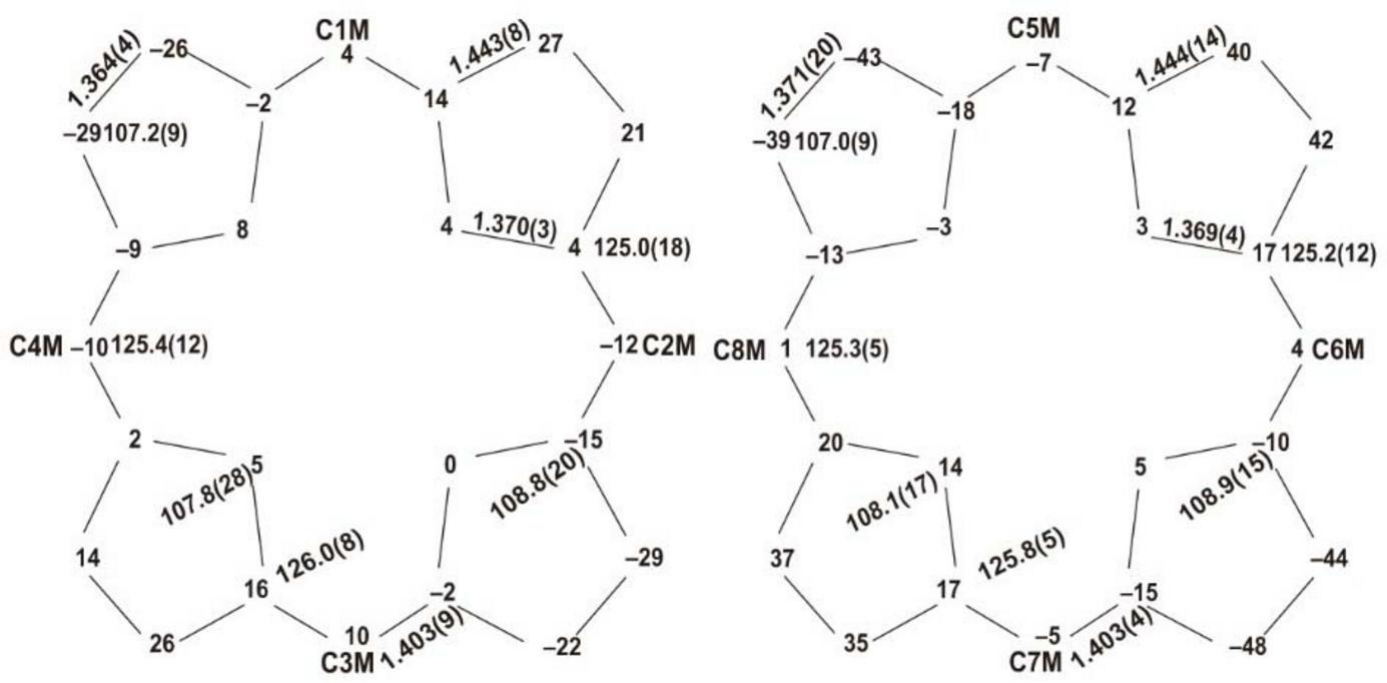
A formal diagram of the porphyrin core of the title compound. Averaged values of the chemically unique bond lengths (Å) and angles (°) are shown. The numbers in parentheses are the e.s.d.’s calculated on the assumption that the averaged values were all drawn from the same population. The perpendicular displacements (in units of 0.01 Å) of the porphyrin core atoms from the 24-atom mean plane are also displayed. Positive numbers indicate a displacement toward the pyridine groups.

The asymmetric unit of the title solvate contains two porphyrin mol­ecules, one disordered *n*-hexane solvate mol­ecule and one ordered *n*-hexane solvate mol­ecule. Figs. 1 ▸ and 2 ▸ graphically represent the mol­ecular structure of the title porphyrin; *n*-hexane (C_6_H_14_) is the lattice solvent, which has been omitted in Fig. 1 ▸. As can be seen, the two porphyrin mol­ecules are alternately embedded together in the asymmetric unit. More qu­anti­tative numerical information is given in Fig. 3 ▸, which contains the detailed displacement of each porphyrin core atom (in units of 0.01 Å) from the 24-atom mean plane. The porphyrin core shows a characteristic saddle-shaped distortion and the maximum deviation from the 24-atom mean plane of the non-hydrogen atoms is 0.48 Å, for atom C12*B*.

**Figure 4:**
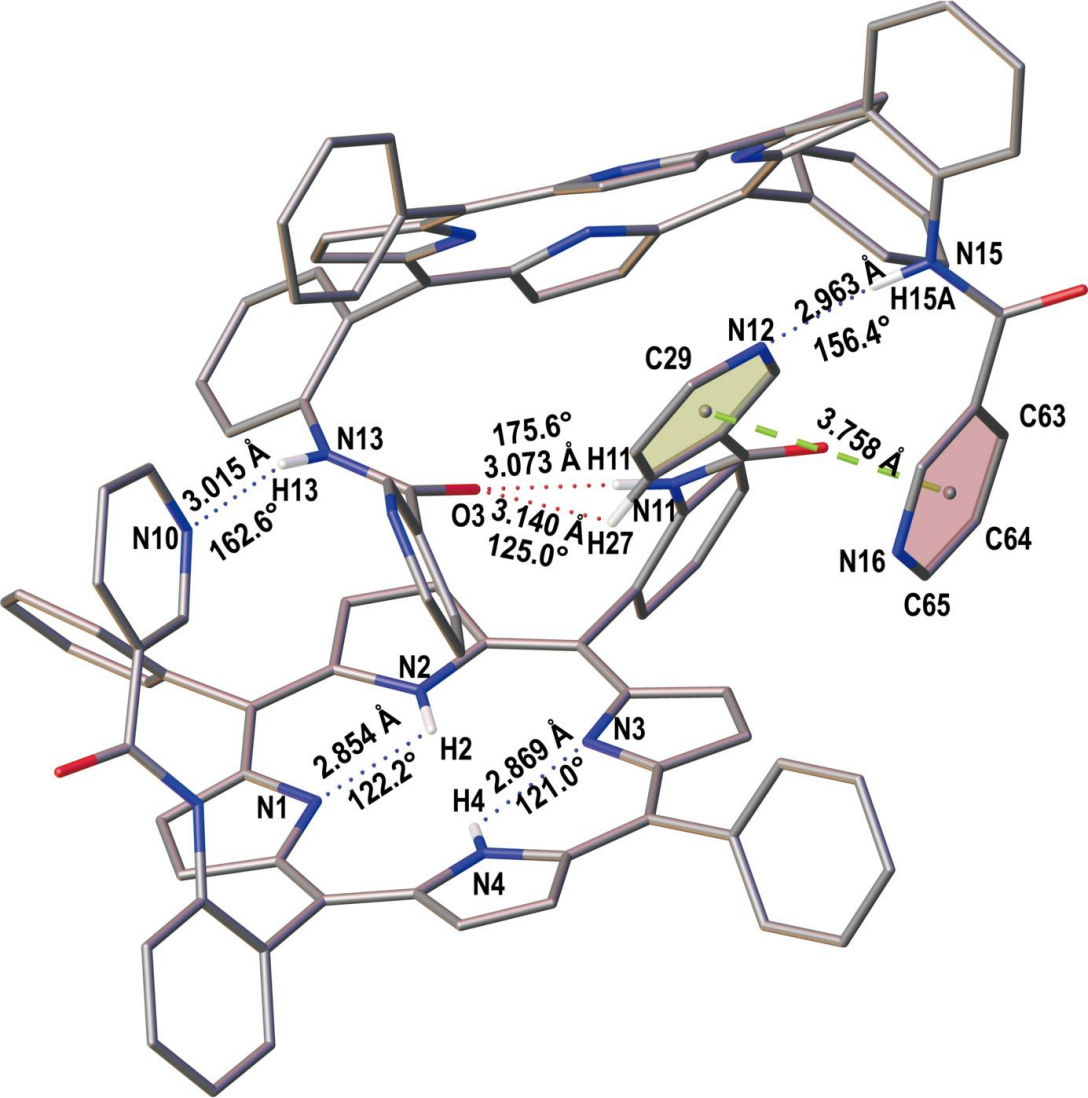
N—H⋯N, N—H⋯O and C—H⋯O hydrogen-bonding inter­actions and relevant inter­molecular π–π inter­actions in the crystal structure of the title compound (dashed lines).

**Figure 5:**
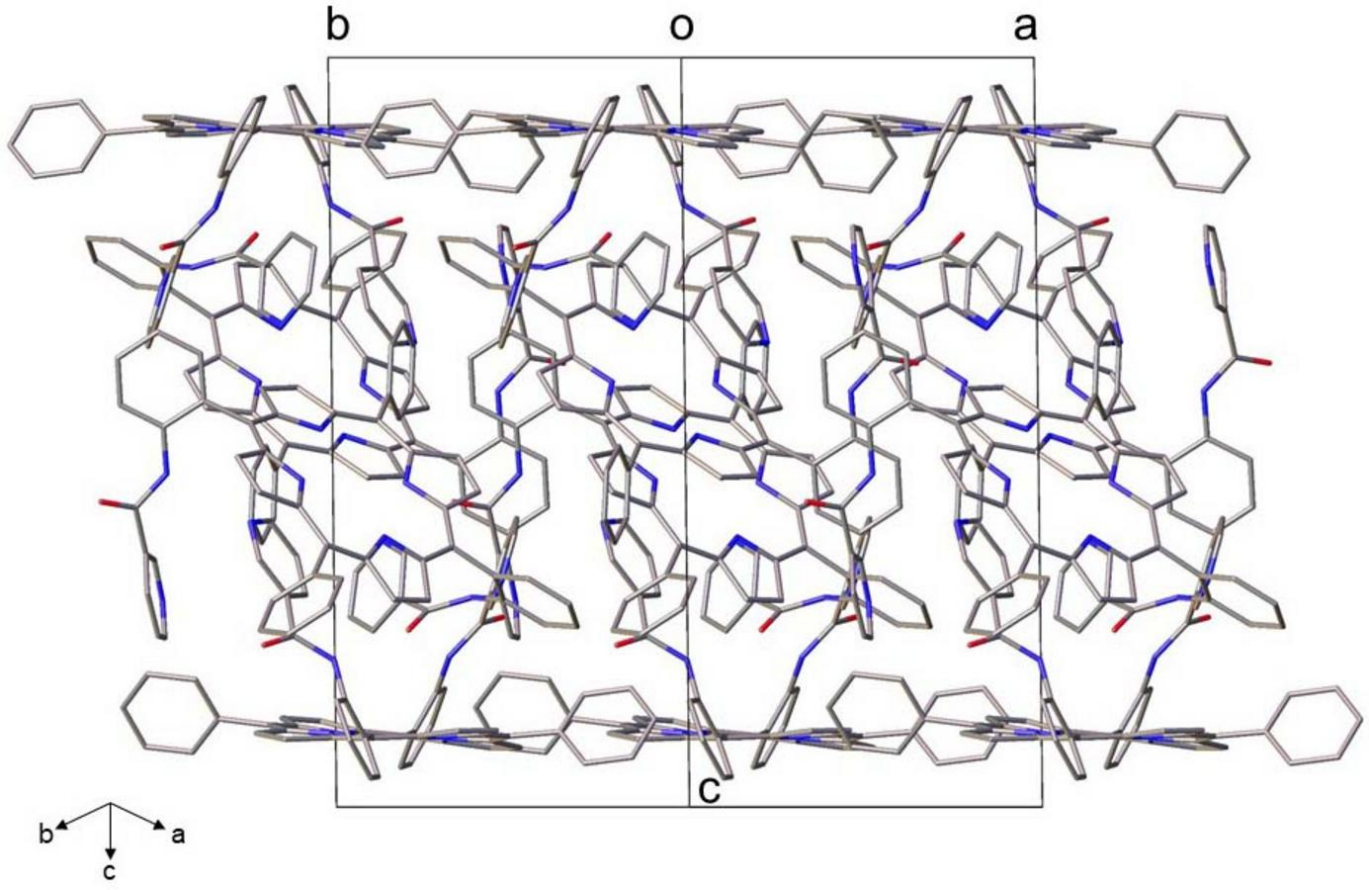
A view of the mol­ecular packing in the crystal structure of the title compound, as seen in a projection along [110]. H atoms and solvent mol­ecules have been omitted for clarity.

In the crystal, N—H⋯N, N—H⋯O and C—H⋯O hydrogen-bonding, as well as π–π inter­actions are found between the two porphyrin mol­ecules, as illustrated in Fig. 4 ▸ and detailed in Table 1 ▸. As can be seen in Fig. 4 ▸, the inter­planar distance between the relevant centroids of the rings in the π–π stacking inter­actions is 3.758 (2) Å, which is consistent with literature data (range 3.3–3.8 Å; Janiak, 2000 ▸; Khavasi & Fard, 2010 ▸). The distance between N10 and N13 is 3.015 (3) Å and the N—H⋯N angle is 163° in the N—H⋯N hydrogen-bonding inter­actions (Fig. 4 ▸, Table 1 ▸). Similar hydrogen-bonding inter­actions are also found between N12 and N15, with a distance of 2.963 (3) Å and an N—H⋯N angle of 156°. All these structural parameters are consistent with literature data where N—H⋯N bonds fall in the range 2.6–3.2 Å, with angles of 120.5–179.7° (Prasad & Govil, 1980 ▸; Aldilla *et al.*, 2017 ▸). Moreover, N—H⋯O and C—H⋯O hydrogen bonds are also found between adjacent porphyrin mol­ecules (Fig. 4 ▸, Table 1 ▸). Furthermore, weak intra­molecular N—H⋯N hydrogen-bonding inter­actions are found in each porphyrin mol­ecule (Fig. 4 ▸, Table 1 ▸). The mol­ecular packing of the title compound is shown in Fig. 5 ▸.

## Synthesis and crystallization

All experimental manipulations were performed under an argon atmosphere using double-manifold vacuum lines, Schlenk vessel and cannula techniques. Except for the solvent used in column chromatography, all solvents used in the experimental process were treated under anhydrous and anaerobic conditions using the pump–freeze–thaw method three times prior to use. Tetra­hydro­furan and *n*-hexane were distilled over CaH_2_ and K—Na alloy, respectively. *αα*-TPP-amino [*meso*-*α,α*-5,15-bis-(2-amino­phen­yl)-10,20-bis-(phen­yl)-porphyrin] and *αα*-*ortho*-amide [*meso*-*α,α*-5,15-bis­(*o*-nicotinamido­phen­yl)-10,20-bis­(phen­yl)porphyrin, that is the title compound] were prepared according to literature protocols (Gotico *et al.*, 2020 ▸; Gunter *et al.*, 1984 ▸), with slight modifications.

Under an argon atmosphere, compound *αα*-TPP-amino (300 mg, 0.46 mmol) was dissolved in anhydrous DCM (25 ml). Nicotinoyl chloride hydro­chloride (202.5 mg, 1.15 mmol) was dissolved in anhydrous pyridine (20 ml) under an Ar atmosphere. Then, the *αα*-TPP-amino solution was slowly added into the pyridine solution and the mixture was refluxed for 30 minutes at 368 K under Ar. After the reaction mixture had cooled to room temperature, silica gel was loaded on the top of silica gel column. The crude product was purified by column chromatography (chloro­form/hexane from 1:3 to 1:0) on silica and finally recrystallized from DCM/MeOH. The solvent was removed under reduced pressure to afford the pure compound *αα*-*ortho*-amide as a purple crystalline solid (216 mg, 55% yield).

To grow single crystals, *αα*-*ortho*-amide (15 mg) was dissolved in 5 ml of tetra­hydro­furan and cannula-transferred into 8 mm glass tubes, then carefully layered with *n*-hexa­nes before sealing the tubes. X-ray quality crystals were obtained after several weeks.

## Refinement

Crystal data, data collection and structure refinement details are summarized in Table 2 ▸. The disordered hexane mol­ecule, C73–C78/C73–C78*A*, occupies two sites with refined occupancies of 0.661 (6) and 0.339 (6). All C atoms in this disordered mol­ecule were restrained to have similar displacement parameters with standard deviation of 0.04 Å^2^, and C74 was restrained to approximate an isotropic behaviour (SIMU and ISOR commands; Sheldrick, 2015*b* ▸). Finally, C—C bond lengths in this mol­ecule were restrained to 1.50 (2) Å (*DFIX* command; Sheldrick, 2015*b* ▸).

## Supplementary Material

Crystal structure: contains datablock(s) I. DOI: 10.1107/S2414314623010854/bh4080sup1.cif

Structure factors: contains datablock(s) I. DOI: 10.1107/S2414314623010854/bh4080Isup2.hkl

CCDC reference: 2320368

Additional supporting information:  crystallographic information; 3D view; checkCIF report

## Figures and Tables

**Figure 2:**
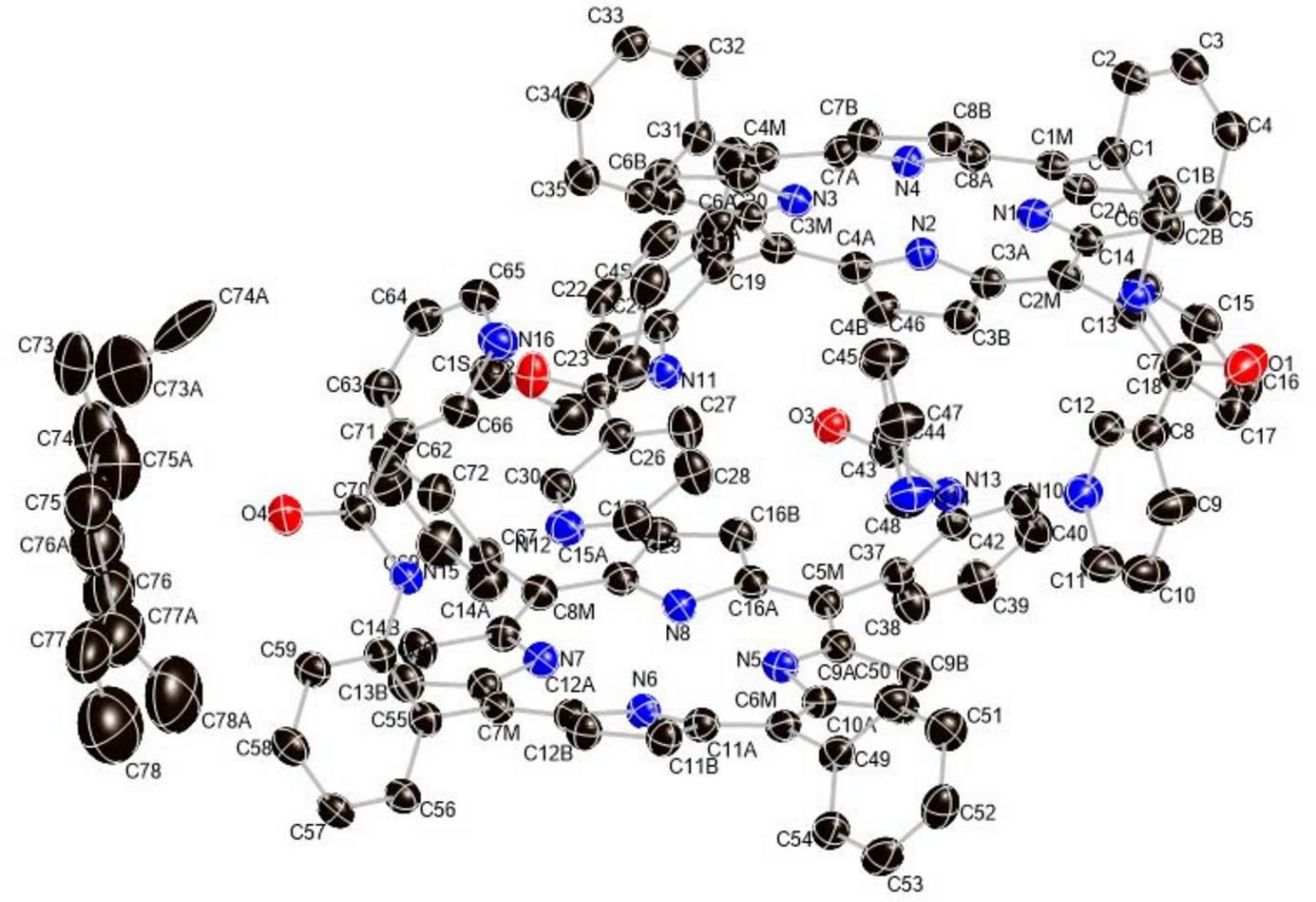
Edge view of the porphyrin complex of the title compound with displacement ellipsoids drawn at the 50% probability level. A disordered *n*-hexane solvate mol­ecule, an ordered *n*-hexane solvate mol­ecule and two porphyrin mol­ecules are shown. Hydrogen atoms are omitted for clarity.

**Table 1:** Hydrogen-bond geometry (Å, °)

*D*—H⋯*A*	*D*—H	H⋯*A*	*D*⋯*A*	*D*—H⋯*A*
N13—H13⋯N10	0.88	2.16	3.015 (3)	163
N15—H15*A*⋯N12	0.88	2.14	2.963 (3)	156
N11—H11⋯O3	0.88	2.19	3.073 (3)	176
C27—H27⋯O3	0.95	2.50	3.140 (4)	125
N2—H2⋯N1	0.88	2.29	2.854 (3)	122
N4—H4⋯N3	0.88	2.31	2.869 (3)	121

**Table 2:** Experimental details

Crystal data	
Chemical formula	C_56_H_38_N_8_O_2_·C_6_H_14_
*M*_r_	941.11
Crystal system, space group	Triclinic, *P* 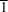
Temperature (K)	100
*a*, *b*, *c* (Å)	13.4628 (2), 17.2828 (3), 21.7586 (4)
α, β, γ (°)	84.624 (2), 84.857 (3), 85.114 (4)
*V* (Å^3^)	5005.05 (15)
*Z*	4
Radiation type	Mo *K*α
μ (mm^−1^)	0.08
Crystal size (mm)	0.68 × 0.36 × 0.12

Data collection	
Diffractometer	Bruker APEXII CCD detector
Absorption correction	Multi-scan (*SADABS*; Krause *et al.*, 2015 ▸)
*T*_min_, *T*_max_	0.972, 0.985
No. of measured, independent and observed [*I* > 2σ(*I*)] reflections	93444, 21131, 14764
*R*_int_	0.054
(sin θ/λ)_max_ (Å^−1^)	0.634

Refinement	
*R*[*F* ^2^ > 2σ(*F* ^2^)], *wR*(*F* ^2^), *S*	0.072, 0.223, 1.07
No. of reflections	21131
No. of parameters	1359
No. of restraints	139
H-atom treatment	H-atom parameters constrained
Δρ_max_, Δρ_min_ (e Å^−3^)	0.68, −0.44

Computer programs: *APEX2* and *SAINT* (Bruker, 2013 ▸), *SHELXT2018/2* (Sheldrick, 2015*a* ▸), *SHELXL2018/3* (Sheldrick, 2015*b* ▸) and *OLEX2* (Dolomanov *et al.*, 2009 ▸).

## full crystallographic data

### Crystal data

**Table:**

C_56_H_38_N_8_O_2_·C_6_H_14_	*Z* = 4
*M**_r_* = 941.11	*F*(000) = 1984
Triclinic, *P*1	*D*_x_ = 1.249 Mg m^−^^3^
*a* = 13.4628 (2) Å	Mo *K*α radiation, λ = 0.71073 Å
*b* = 17.2828 (3) Å	Cell parameters from 1983 reflections
*c* = 21.7586 (4) Å	θ = 3–23°
α = 84.624 (2)°	µ = 0.08 mm^−^^1^
β = 84.857 (3)°	*T* = 100 K
γ = 85.114 (4)°	Block, black
*V* = 5005.05 (15) Å^3^	0.68 × 0.36 × 0.12 mm

### Data collection

**Table:**

Bruker APEXII CCD detector diffractometer	14764 reflections with *I* > 2σ(*I*)
Radiation source: fine-focus sealed X-ray tube	*R*_int_ = 0.054
phi and ω scans	θ_max_ = 26.8°, θ_min_ = 1.9°
Absorption correction: multi-scan (SADABS; Krause *et al.*, 2015)	*h* = −17→16
*T*_min_ = 0.972, *T*_max_ = 0.985	*k* = −21→21
93444 measured reflections	*l* = −27→27
21131 independent reflections	

### Refinement

**Table:**

Refinement on *F*^2^	Hydrogen site location: inferred from neighbouring sites
Least-squares matrix: full	H-atom parameters constrained
*R*[*F*^2^ > 2σ(*F*^2^)] = 0.072	*w* = 1/[σ^2^(*F*_o_^2^) + (0.0964*P*)^2^ + 6.4713*P*] where *P* = (*F*_o_^2^ + 2*F*_c_^2^)/3
*wR*(*F*^2^) = 0.223	(Δ/σ)_max_ = 0.005
*S* = 1.07	Δρ_max_ = 0.68 e Å^−^^3^
21131 reflections	Δρ_min_ = −0.44 e Å^−^^3^
1359 parameters	Extinction correction: *SHELXL2018/3* (Sheldrick 2015b), Fc^*^=kFc[1+0.001xFc^2^λ^3^/sin(2θ)]^-1/4^
139 restraints	Extinction coefficient: 0.0090 (7)
Primary atom site location: dual	

### Fractional atomic coordinates and isotropic or equivalent isotropic displacement parameters (Å^2^)

**Table:**

	*x*	*y*	*z*	*U*_iso_*/*U*_eq_	Occ. (<1)
O1	0.20692 (19)	−0.26886 (14)	0.74653 (12)	0.0560 (7)	
O2	0.28928 (17)	0.48913 (12)	0.78378 (11)	0.0441 (5)	
N1	0.28521 (16)	−0.02538 (13)	0.90787 (10)	0.0292 (5)	
N2	0.38928 (16)	0.11200 (12)	0.90651 (10)	0.0275 (5)	
H2	0.326732	0.099921	0.909537	0.033*	
N3	0.19286 (16)	0.20406 (13)	0.90194 (10)	0.0283 (5)	
N4	0.08843 (16)	0.06595 (12)	0.90299 (10)	0.0267 (4)	
H4	0.148173	0.076833	0.911402	0.032*	
N9	0.16245 (17)	−0.16196 (14)	0.79927 (11)	0.0337 (5)	
H9	0.171415	−0.112056	0.798836	0.040*	
N10	0.41409 (19)	−0.06175 (15)	0.69702 (13)	0.0412 (6)	
N11	0.36518 (16)	0.36762 (13)	0.80368 (11)	0.0304 (5)	
H11	0.376070	0.320692	0.790700	0.036*	
N12	0.19251 (18)	0.42752 (15)	0.62009 (12)	0.0388 (6)	
C1	0.07139 (19)	−0.15063 (15)	0.89955 (12)	0.0282 (5)	
C1A	0.2236 (2)	−0.08503 (16)	0.91206 (12)	0.0298 (6)	
C1B	0.2799 (2)	−0.15989 (16)	0.92083 (13)	0.0336 (6)	
H1B	0.253911	−0.209706	0.925963	0.040*	
C1M	0.12049 (19)	−0.07745 (15)	0.90475 (12)	0.0273 (5)	
C2	0.00369 (19)	−0.17914 (16)	0.94637 (13)	0.0309 (6)	
H2A	−0.013499	−0.150688	0.981611	0.037*	
C2A	0.37977 (19)	−0.06083 (16)	0.91112 (12)	0.0297 (6)	
C2B	0.3773 (2)	−0.14466 (16)	0.92024 (14)	0.0339 (6)	
H2B	0.432876	−0.181793	0.924899	0.041*	
C2M	0.46878 (19)	−0.02175 (16)	0.90519 (12)	0.0291 (5)	
C3	−0.0393 (2)	−0.24861 (17)	0.94251 (14)	0.0349 (6)	
H3	−0.084073	−0.268276	0.975399	0.042*	
C3A	0.47157 (19)	0.05909 (16)	0.90265 (12)	0.0292 (5)	
C3B	0.5583 (2)	0.10319 (16)	0.89737 (13)	0.0322 (6)	
H3B	0.625985	0.082361	0.893811	0.039*	
C3M	0.35797 (19)	0.25477 (15)	0.90753 (12)	0.0279 (5)	
C4	−0.0167 (2)	−0.28865 (17)	0.89081 (14)	0.0356 (6)	
H4A	−0.046369	−0.336038	0.888101	0.043*	
C4A	0.41866 (19)	0.18596 (16)	0.90497 (12)	0.0282 (5)	
C4B	0.5264 (2)	0.18020 (17)	0.89837 (13)	0.0322 (6)	
H4B	0.567832	0.222453	0.895276	0.039*	
C4M	0.01238 (19)	0.20094 (15)	0.89042 (12)	0.0271 (5)	
C5	0.0489 (2)	−0.26087 (17)	0.84240 (14)	0.0354 (6)	
H5	0.062846	−0.288448	0.806449	0.042*	
C5A	0.25276 (19)	0.26280 (15)	0.90722 (12)	0.0273 (5)	
C5B	0.1943 (2)	0.33718 (16)	0.90920 (13)	0.0319 (6)	
H5B	0.218209	0.385978	0.914847	0.038*	
C6	0.0941 (2)	−0.19238 (16)	0.84696 (13)	0.0307 (6)	
C6A	0.09854 (19)	0.23946 (15)	0.89743 (12)	0.0276 (5)	
C6B	0.0981 (2)	0.32313 (16)	0.90133 (13)	0.0323 (6)	
H6B	0.042191	0.360420	0.898895	0.039*	
C7	0.2156 (2)	−0.20076 (17)	0.75427 (14)	0.0379 (7)	
C7A	0.00995 (19)	0.12013 (15)	0.89219 (12)	0.0284 (5)	
C7B	−0.0731 (2)	0.07808 (16)	0.88126 (13)	0.0308 (6)	
H7B	−0.137964	0.100301	0.872869	0.037*	
C8	0.2908 (2)	−0.15584 (17)	0.71382 (14)	0.0373 (6)	
C8A	0.06013 (19)	−0.00779 (15)	0.89875 (12)	0.0276 (5)	
C8B	−0.04260 (19)	0.00044 (16)	0.88500 (13)	0.0309 (6)	
H8B	−0.082434	−0.040782	0.879465	0.037*	
C9	0.3117 (3)	−0.1759 (2)	0.65345 (16)	0.0524 (9)	
H9A	0.278128	−0.215814	0.638734	0.063*	
C10	0.3823 (3)	−0.1371 (2)	0.61505 (18)	0.0580 (10)	
H10	0.396882	−0.148762	0.573238	0.070*	
C11	0.4312 (2)	−0.0808 (2)	0.63888 (16)	0.0463 (8)	
H11A	0.479690	−0.054245	0.612345	0.056*	
C12	0.3433 (2)	−0.09821 (17)	0.73352 (14)	0.0357 (6)	
H12	0.328536	−0.083909	0.774642	0.043*	
C13	0.56577 (19)	−0.06946 (15)	0.89936 (13)	0.0294 (6)	
C14	0.6381 (2)	−0.06634 (18)	0.94136 (14)	0.0358 (6)	
H14	0.624396	−0.035205	0.975311	0.043*	
C15	0.7304 (2)	−0.10908 (19)	0.93320 (15)	0.0408 (7)	
H15	0.778831	−0.108209	0.962281	0.049*	
C16	0.7514 (2)	−0.15253 (18)	0.88300 (16)	0.0412 (7)	
H16	0.815108	−0.180046	0.876881	0.049*	
C17	0.6806 (2)	−0.15641 (17)	0.84157 (15)	0.0384 (7)	
H17	0.695454	−0.186913	0.807312	0.046*	
C18	0.5875 (2)	−0.11573 (16)	0.84992 (14)	0.0329 (6)	
H18	0.538429	−0.119440	0.821809	0.039*	
C19	0.41029 (19)	0.32758 (15)	0.90788 (13)	0.0289 (5)	
C20	0.4598 (2)	0.34107 (17)	0.95927 (13)	0.0337 (6)	
H20	0.461281	0.302972	0.993682	0.040*	
C21	0.5067 (2)	0.40921 (18)	0.96076 (15)	0.0378 (7)	
H21	0.539976	0.417745	0.995930	0.045*	
C22	0.5045 (2)	0.46470 (17)	0.91056 (14)	0.0360 (6)	
H22	0.536259	0.511548	0.911568	0.043*	
C23	0.4567 (2)	0.45270 (16)	0.85900 (14)	0.0337 (6)	
H23	0.455051	0.491398	0.825013	0.040*	
C24	0.41075 (19)	0.38334 (16)	0.85698 (13)	0.0300 (6)	
C25	0.3052 (2)	0.42167 (16)	0.77147 (13)	0.0328 (6)	
C26	0.2594 (2)	0.39223 (17)	0.71847 (13)	0.0337 (6)	
C27	0.2354 (2)	0.31646 (18)	0.71677 (15)	0.0422 (7)	
H27	0.248323	0.278479	0.750148	0.051*	
C28	0.1921 (2)	0.29670 (19)	0.66547 (16)	0.0445 (7)	
H28	0.175339	0.244850	0.663058	0.053*	
C29	0.1738 (2)	0.35356 (19)	0.61790 (15)	0.0402 (7)	
H29	0.146691	0.339182	0.582148	0.048*	
C30	0.2353 (2)	0.44600 (17)	0.66957 (13)	0.0350 (6)	
H30	0.249823	0.498557	0.671216	0.042*	
C31	−0.08318 (19)	0.24882 (15)	0.88204 (12)	0.0284 (5)	
C32	−0.16413 (19)	0.24174 (16)	0.92646 (13)	0.0311 (6)	
H32	−0.158074	0.205753	0.961953	0.037*	
C33	−0.2535 (2)	0.28688 (17)	0.91924 (13)	0.0332 (6)	
H33	−0.307670	0.282390	0.950172	0.040*	
C34	−0.2638 (2)	0.33832 (16)	0.86715 (14)	0.0332 (6)	
H34	−0.325399	0.368292	0.861887	0.040*	
C35	−0.1837 (2)	0.34616 (17)	0.82240 (14)	0.0351 (6)	
H35	−0.190418	0.382009	0.786883	0.042*	
C36	−0.0946 (2)	0.30176 (16)	0.82970 (13)	0.0329 (6)	
H36	−0.040390	0.307126	0.798914	0.039*	
O3	0.41181 (15)	0.20173 (12)	0.76351 (9)	0.0367 (5)	
O4	0.02592 (19)	0.68289 (13)	0.59252 (11)	0.0498 (6)	
N5	0.45989 (16)	0.25411 (14)	0.56725 (10)	0.0311 (5)	
N6	0.33536 (17)	0.36793 (13)	0.49578 (11)	0.0309 (5)	
H6	0.385456	0.370552	0.518657	0.037*	
N7	0.40371 (17)	0.49063 (14)	0.56374 (11)	0.0325 (5)	
N8	0.51871 (16)	0.37442 (13)	0.63945 (11)	0.0306 (5)	
H8	0.487787	0.374346	0.605495	0.037*	
N13	0.48415 (17)	0.09330 (14)	0.72012 (11)	0.0331 (5)	
H13	0.471537	0.049545	0.705740	0.040*	
N14	0.2125 (2)	0.07452 (17)	0.64414 (12)	0.0452 (6)	
N15	0.10001 (18)	0.57038 (14)	0.55515 (11)	0.0347 (5)	
H15A	0.119644	0.521536	0.566048	0.042*	
N16	−0.0269 (2)	0.45926 (15)	0.72479 (12)	0.0421 (6)	
C5M	0.5698 (2)	0.23340 (16)	0.65311 (13)	0.0309 (6)	
C6M	0.3568 (2)	0.22667 (16)	0.48453 (12)	0.0313 (6)	
C7M	0.2771 (2)	0.50764 (16)	0.48777 (13)	0.0313 (6)	
C8M	0.4987 (2)	0.51638 (16)	0.65029 (13)	0.0322 (6)	
C9A	0.5274 (2)	0.20977 (16)	0.60165 (13)	0.0304 (6)	
C9B	0.5461 (2)	0.13330 (16)	0.57942 (13)	0.0327 (6)	
H9B	0.591438	0.092353	0.594644	0.039*	
C10A	0.4311 (2)	0.20651 (16)	0.52588 (13)	0.0315 (6)	
C10B	0.4864 (2)	0.13111 (16)	0.53259 (13)	0.0325 (6)	
H10B	0.481842	0.088333	0.508833	0.039*	
C11A	0.3090 (2)	0.30137 (16)	0.47430 (13)	0.0325 (6)	
C11B	0.2232 (2)	0.32158 (17)	0.43988 (14)	0.0359 (6)	
H11B	0.188712	0.286984	0.419521	0.043*	
C12A	0.2724 (2)	0.42972 (17)	0.47642 (13)	0.0327 (6)	
C12B	0.2001 (2)	0.40005 (17)	0.44183 (14)	0.0356 (6)	
H12B	0.145881	0.429646	0.423556	0.043*	
C13A	0.3426 (2)	0.53557 (16)	0.52548 (13)	0.0319 (6)	
C13B	0.3488 (2)	0.61832 (18)	0.53281 (15)	0.0386 (7)	
H13B	0.316120	0.661356	0.510273	0.046*	
C14A	0.4432 (2)	0.53971 (16)	0.59915 (13)	0.0331 (6)	
C14B	0.4128 (2)	0.62123 (18)	0.57976 (15)	0.0406 (7)	
H14B	0.432351	0.666455	0.595686	0.049*	
C15A	0.53066 (19)	0.43930 (16)	0.66930 (13)	0.0314 (6)	
C15B	0.5832 (2)	0.41338 (17)	0.72288 (13)	0.0326 (6)	
H15B	0.601718	0.445570	0.752364	0.039*	
C16A	0.56262 (19)	0.30978 (16)	0.67095 (13)	0.0306 (6)	
C16B	0.6018 (2)	0.33495 (17)	0.72412 (13)	0.0324 (6)	
H16B	0.635019	0.302438	0.754839	0.039*	
C37	0.6287 (2)	0.17261 (16)	0.69120 (13)	0.0323 (6)	
C38	0.7302 (2)	0.17898 (18)	0.69539 (15)	0.0408 (7)	
H38	0.760235	0.223082	0.674727	0.049*	
C39	0.7891 (2)	0.12305 (19)	0.72874 (16)	0.0439 (7)	
H39	0.858132	0.129040	0.730889	0.053*	
C40	0.7458 (2)	0.05834 (18)	0.75887 (14)	0.0416 (7)	
H40	0.785338	0.019417	0.781504	0.050*	
C41	0.6454 (2)	0.05065 (17)	0.75589 (13)	0.0375 (6)	
H41	0.616114	0.006234	0.776608	0.045*	
C42	0.5861 (2)	0.10705 (16)	0.72296 (12)	0.0310 (6)	
C43	0.4050 (2)	0.14195 (16)	0.73771 (13)	0.0327 (6)	
C44	0.3069 (2)	0.11713 (17)	0.72196 (13)	0.0337 (6)	
C45	0.2245 (2)	0.11539 (18)	0.76461 (14)	0.0391 (7)	
H45	0.227959	0.130021	0.805327	0.047*	
C46	0.1365 (2)	0.0917 (2)	0.74634 (15)	0.0452 (8)	
H46	0.078537	0.089437	0.774586	0.054*	
C47	0.1342 (2)	0.0714 (2)	0.68670 (15)	0.0456 (8)	
H47	0.073802	0.054312	0.675151	0.055*	
C48	0.2965 (2)	0.09689 (19)	0.66259 (14)	0.0395 (7)	
H48	0.353094	0.099169	0.633247	0.047*	
C49	0.3256 (2)	0.16322 (17)	0.44961 (13)	0.0337 (6)	
C50	0.2865 (2)	0.09738 (18)	0.48121 (15)	0.0408 (7)	
H50	0.276891	0.093523	0.525149	0.049*	
C51	0.2613 (3)	0.0372 (2)	0.44886 (16)	0.0476 (8)	
H51	0.234930	−0.007648	0.470846	0.057*	
C52	0.2743 (3)	0.0422 (2)	0.38476 (16)	0.0453 (7)	
H52	0.257627	0.000763	0.362804	0.054*	
C53	0.3116 (3)	0.1076 (2)	0.35316 (15)	0.0451 (8)	
H53	0.319754	0.111563	0.309182	0.054*	
C54	0.3376 (2)	0.16820 (18)	0.38514 (14)	0.0397 (7)	
H54	0.363506	0.213033	0.362874	0.048*	
C55	0.2033 (2)	0.56619 (16)	0.45784 (13)	0.0313 (6)	
C56	0.2177 (2)	0.58926 (16)	0.39491 (13)	0.0329 (6)	
H56	0.273651	0.566664	0.371239	0.039*	
C57	0.1526 (2)	0.64436 (17)	0.36595 (13)	0.0352 (6)	
H57	0.163659	0.659547	0.322936	0.042*	
C58	0.0711 (2)	0.67700 (17)	0.40072 (14)	0.0368 (6)	
H58	0.026601	0.715549	0.381533	0.044*	
C59	0.0540 (2)	0.65387 (17)	0.46333 (14)	0.0359 (6)	
H59	−0.002486	0.676342	0.486592	0.043*	
C60	0.1191 (2)	0.59786 (16)	0.49229 (13)	0.0317 (6)	
C61	0.0534 (2)	0.61377 (17)	0.60037 (14)	0.0360 (6)	
C62	0.0372 (2)	0.56957 (17)	0.66253 (14)	0.0350 (6)	
C63	0.0687 (2)	0.59759 (18)	0.71437 (14)	0.0413 (7)	
H63	0.099459	0.645439	0.711058	0.050*	
C64	0.0548 (3)	0.55498 (19)	0.77122 (15)	0.0439 (7)	
H64	0.078046	0.571785	0.807345	0.053*	
C65	0.0065 (3)	0.48781 (19)	0.77388 (15)	0.0440 (7)	
H65	−0.004028	0.459464	0.813160	0.053*	
C66	−0.0093 (2)	0.49965 (17)	0.66982 (14)	0.0364 (6)	
H66	−0.029462	0.479759	0.634022	0.044*	
C67	0.5204 (2)	0.57794 (16)	0.69036 (14)	0.0346 (6)	
C68	0.6176 (2)	0.59663 (19)	0.69528 (16)	0.0432 (7)	
H68	0.671692	0.571871	0.671458	0.052*	
C69	0.6361 (3)	0.6515 (2)	0.73497 (18)	0.0517 (8)	
H69	0.702800	0.663425	0.738479	0.062*	
C70	0.5583 (3)	0.6885 (2)	0.76919 (17)	0.0512 (8)	
H70	0.571353	0.725853	0.796198	0.061*	
C71	0.4607 (3)	0.67115 (19)	0.76417 (16)	0.0454 (7)	
H71	0.406889	0.696914	0.787491	0.054*	
C72	0.4419 (2)	0.61601 (18)	0.72493 (15)	0.0406 (7)	
H72	0.375045	0.604157	0.721648	0.049*	
C1S	0.2702 (3)	0.3745 (2)	1.09131 (18)	0.0536 (9)	
H1SA	0.327862	0.389810	1.063473	0.080*	
H1SB	0.229600	0.342706	1.069987	0.080*	
H1SC	0.293620	0.344234	1.128325	0.080*	
C2S	0.2081 (3)	0.4462 (2)	1.10992 (18)	0.0532 (9)	
H2SA	0.251318	0.479834	1.128310	0.064*	
H2SB	0.155587	0.430359	1.142351	0.064*	
C3S	0.1579 (3)	0.4940 (2)	1.05635 (18)	0.0522 (8)	
H3SA	0.114315	0.460567	1.038025	0.063*	
H3SB	0.114852	0.537800	1.073084	0.063*	
C4S	0.2326 (2)	0.5267 (2)	1.00537 (16)	0.0457 (8)	
H4SA	0.280346	0.555697	1.024501	0.055*	
H4SB	0.271101	0.482665	0.986013	0.055*	
C5S	0.1850 (3)	0.5804 (2)	0.95516 (19)	0.0592 (10)	
H5SA	0.139100	0.550964	0.934851	0.071*	
H5SB	0.144617	0.623587	0.974533	0.071*	
C6S	0.2599 (3)	0.6141 (2)	0.90661 (19)	0.0586 (9)	
H6SA	0.224714	0.648492	0.875855	0.088*	
H6SB	0.298727	0.571760	0.886162	0.088*	
H6SC	0.305117	0.643989	0.926221	0.088*	
C73	0.9500 (5)	−0.1062 (4)	0.7118 (4)	0.0697 (19)	0.661 (6)
H73A	0.907803	−0.149970	0.720667	0.105*	0.661 (6)
H73B	0.908715	−0.058926	0.699036	0.105*	0.661 (6)
H73C	0.982295	−0.097995	0.749008	0.105*	0.661 (6)
C73A	0.9390 (12)	−0.1610 (11)	0.7002 (9)	0.108 (4)	0.339 (6)
H73D	0.970845	−0.151645	0.737837	0.129*	0.339 (6)
H73E	0.885282	−0.118601	0.695686	0.129*	0.339 (6)
C74	1.0221 (10)	−0.1224 (8)	0.6648 (6)	0.110 (3)	0.661 (6)
H74A	1.054415	−0.175066	0.675192	0.131*	0.661 (6)
H74B	1.073933	−0.084928	0.664015	0.131*	0.661 (6)
C74A	0.8803 (8)	−0.2345 (9)	0.7214 (5)	0.085 (4)	0.339 (6)
H74C	0.837263	−0.243603	0.689193	0.127*	0.339 (6)
H74D	0.839090	−0.225874	0.759947	0.127*	0.339 (6)
H74E	0.927827	−0.280038	0.728278	0.127*	0.339 (6)
C75	0.9867 (7)	−0.1198 (6)	0.5980 (6)	0.094 (2)	0.661 (6)
H75A	0.941776	−0.162116	0.597431	0.112*	0.661 (6)
H75B	0.946482	−0.069809	0.589807	0.112*	0.661 (6)
C75A	1.026 (2)	−0.1366 (17)	0.6422 (10)	0.107 (3)	0.339 (6)
H75C	1.049550	−0.087125	0.652651	0.129*	0.339 (6)
H75D	1.082615	−0.176323	0.646990	0.129*	0.339 (6)
C76	1.0697 (9)	−0.1278 (6)	0.5438 (6)	0.111 (3)	0.661 (6)
H76A	1.109469	−0.178372	0.549926	0.133*	0.661 (6)
H76B	1.115355	−0.085605	0.542919	0.133*	0.661 (6)
C76A	1.014 (2)	−0.1251 (15)	0.5719 (10)	0.113 (3)	0.339 (6)
H76C	0.989314	−0.069490	0.566026	0.136*	0.339 (6)
H76D	0.955074	−0.154316	0.568063	0.136*	0.339 (6)
C77	1.0153 (8)	−0.1221 (5)	0.4749 (6)	0.114 (3)	0.661 (6)
H77A	0.970949	−0.164794	0.474071	0.137*	0.661 (6)
H77B	0.976451	−0.071428	0.467163	0.137*	0.661 (6)
C77A	1.0801 (17)	−0.1377 (12)	0.5074 (13)	0.109 (3)	0.339 (6)
H77C	1.033530	−0.162942	0.484213	0.131*	0.339 (6)
H77D	1.080913	−0.083806	0.487604	0.131*	0.339 (6)
C78	1.0968 (10)	−0.1297 (7)	0.4326 (7)	0.157 (4)	0.661 (6)
H78A	1.126118	−0.079460	0.423811	0.235*	0.661 (6)
H78B	1.075874	−0.146431	0.394272	0.235*	0.661 (6)
H78C	1.146625	−0.168661	0.449606	0.235*	0.661 (6)
C78A	1.1851 (15)	−0.1756 (11)	0.4823 (12)	0.133 (6)	0.339 (6)
H78D	1.176972	−0.203559	0.446128	0.199*	0.339 (6)
H78E	1.212709	−0.212176	0.514797	0.199*	0.339 (6)
H78F	1.230899	−0.134598	0.470416	0.199*	0.339 (6)

### Atomic displacement parameters (Å^2^)

**Table:**

	*U*^11^	*U*^22^	*U*^33^	*U*^12^	*U*^13^	*U*^23^
O1	0.0569 (14)	0.0430 (13)	0.0682 (16)	−0.0171 (11)	0.0221 (12)	−0.0203 (12)
O2	0.0474 (12)	0.0318 (11)	0.0545 (14)	0.0014 (9)	−0.0130 (10)	−0.0065 (10)
N1	0.0249 (11)	0.0331 (12)	0.0295 (11)	−0.0029 (9)	−0.0023 (9)	−0.0014 (9)
N2	0.0231 (10)	0.0282 (11)	0.0310 (11)	−0.0023 (8)	−0.0018 (9)	−0.0018 (9)
N3	0.0246 (10)	0.0314 (12)	0.0289 (11)	−0.0038 (9)	−0.0025 (9)	−0.0016 (9)
N4	0.0236 (10)	0.0277 (11)	0.0293 (11)	−0.0023 (8)	−0.0040 (8)	−0.0036 (9)
N9	0.0359 (12)	0.0288 (12)	0.0361 (13)	−0.0084 (9)	0.0070 (10)	−0.0047 (10)
N10	0.0389 (13)	0.0386 (14)	0.0462 (15)	−0.0087 (11)	0.0064 (11)	−0.0080 (11)
N11	0.0299 (11)	0.0284 (11)	0.0332 (12)	−0.0027 (9)	−0.0016 (9)	−0.0048 (9)
N12	0.0351 (13)	0.0440 (15)	0.0357 (13)	−0.0011 (11)	−0.0010 (10)	0.0025 (11)
C1	0.0243 (12)	0.0284 (13)	0.0310 (13)	−0.0021 (10)	−0.0026 (10)	0.0017 (10)
C1A	0.0291 (13)	0.0314 (14)	0.0283 (13)	−0.0023 (10)	−0.0003 (10)	−0.0008 (11)
C1B	0.0311 (14)	0.0288 (14)	0.0395 (15)	−0.0024 (11)	−0.0019 (11)	0.0034 (11)
C1M	0.0252 (12)	0.0309 (13)	0.0254 (12)	−0.0044 (10)	0.0009 (10)	−0.0009 (10)
C2	0.0260 (12)	0.0374 (15)	0.0290 (13)	−0.0029 (11)	−0.0046 (10)	0.0016 (11)
C2A	0.0279 (13)	0.0316 (14)	0.0289 (13)	−0.0028 (10)	−0.0011 (10)	0.0016 (11)
C2B	0.0272 (13)	0.0320 (14)	0.0410 (16)	−0.0009 (11)	−0.0026 (11)	0.0037 (12)
C2M	0.0267 (13)	0.0327 (14)	0.0272 (13)	−0.0022 (10)	−0.0028 (10)	0.0018 (10)
C3	0.0280 (13)	0.0393 (16)	0.0366 (15)	−0.0073 (11)	−0.0043 (11)	0.0060 (12)
C3A	0.0260 (12)	0.0336 (14)	0.0278 (13)	−0.0008 (10)	−0.0033 (10)	−0.0012 (11)
C3B	0.0266 (13)	0.0339 (14)	0.0360 (15)	−0.0023 (11)	−0.0004 (11)	−0.0038 (11)
C3M	0.0266 (12)	0.0323 (14)	0.0251 (12)	−0.0052 (10)	−0.0019 (10)	−0.0021 (10)
C4	0.0299 (14)	0.0311 (14)	0.0455 (17)	−0.0071 (11)	−0.0026 (12)	0.0016 (12)
C4A	0.0252 (12)	0.0324 (14)	0.0275 (13)	−0.0042 (10)	−0.0015 (10)	−0.0040 (10)
C4B	0.0264 (13)	0.0363 (15)	0.0346 (14)	−0.0041 (11)	−0.0030 (11)	−0.0043 (11)
C4M	0.0250 (12)	0.0307 (13)	0.0253 (12)	−0.0005 (10)	−0.0015 (10)	−0.0026 (10)
C5	0.0329 (14)	0.0322 (15)	0.0415 (16)	−0.0056 (11)	0.0016 (12)	−0.0073 (12)
C5A	0.0270 (12)	0.0282 (13)	0.0265 (13)	−0.0029 (10)	−0.0016 (10)	−0.0010 (10)
C5B	0.0293 (13)	0.0315 (14)	0.0348 (14)	−0.0043 (11)	−0.0015 (11)	−0.0019 (11)
C6	0.0279 (13)	0.0288 (14)	0.0347 (14)	−0.0048 (10)	0.0009 (11)	0.0007 (11)
C6A	0.0247 (12)	0.0302 (13)	0.0279 (13)	−0.0024 (10)	−0.0015 (10)	−0.0024 (10)
C6B	0.0288 (13)	0.0331 (14)	0.0345 (14)	−0.0013 (11)	0.0007 (11)	−0.0038 (11)
C7	0.0359 (15)	0.0363 (16)	0.0427 (17)	−0.0093 (12)	0.0031 (12)	−0.0099 (13)
C7A	0.0253 (12)	0.0309 (14)	0.0287 (13)	−0.0006 (10)	−0.0006 (10)	−0.0040 (10)
C7B	0.0251 (12)	0.0338 (14)	0.0338 (14)	−0.0012 (10)	−0.0054 (10)	−0.0027 (11)
C8	0.0364 (15)	0.0361 (15)	0.0395 (16)	−0.0059 (12)	0.0046 (12)	−0.0080 (12)
C8A	0.0259 (12)	0.0301 (13)	0.0265 (13)	−0.0022 (10)	−0.0016 (10)	−0.0017 (10)
C8B	0.0265 (13)	0.0340 (14)	0.0325 (14)	−0.0057 (10)	−0.0019 (10)	−0.0028 (11)
C9	0.055 (2)	0.059 (2)	0.0465 (19)	−0.0234 (17)	0.0133 (16)	−0.0226 (16)
C10	0.063 (2)	0.066 (2)	0.047 (2)	−0.0238 (19)	0.0187 (17)	−0.0188 (17)
C11	0.0414 (17)	0.0495 (19)	0.0471 (18)	−0.0124 (14)	0.0129 (14)	−0.0069 (15)
C12	0.0342 (14)	0.0333 (15)	0.0392 (16)	−0.0065 (11)	0.0047 (12)	−0.0044 (12)
C13	0.0270 (13)	0.0280 (13)	0.0323 (14)	−0.0014 (10)	−0.0050 (10)	0.0037 (11)
C14	0.0296 (14)	0.0428 (16)	0.0338 (15)	−0.0017 (12)	−0.0045 (11)	0.0031 (12)
C15	0.0316 (14)	0.0481 (18)	0.0417 (17)	0.0017 (13)	−0.0096 (12)	0.0038 (14)
C16	0.0291 (14)	0.0371 (16)	0.0551 (19)	0.0040 (12)	−0.0036 (13)	0.0017 (14)
C17	0.0345 (15)	0.0337 (15)	0.0459 (17)	−0.0003 (12)	−0.0003 (13)	−0.0035 (13)
C18	0.0299 (13)	0.0285 (14)	0.0402 (15)	−0.0028 (10)	−0.0050 (11)	−0.0003 (11)
C19	0.0231 (12)	0.0298 (13)	0.0346 (14)	−0.0022 (10)	−0.0005 (10)	−0.0094 (11)
C20	0.0297 (13)	0.0384 (15)	0.0337 (14)	0.0006 (11)	−0.0036 (11)	−0.0090 (12)
C21	0.0280 (13)	0.0442 (17)	0.0437 (17)	−0.0019 (12)	−0.0044 (12)	−0.0161 (13)
C22	0.0293 (14)	0.0354 (15)	0.0455 (17)	−0.0050 (11)	0.0002 (12)	−0.0161 (13)
C23	0.0296 (13)	0.0313 (14)	0.0404 (15)	−0.0042 (11)	0.0019 (11)	−0.0069 (12)
C24	0.0247 (12)	0.0325 (14)	0.0335 (14)	−0.0031 (10)	−0.0006 (10)	−0.0081 (11)
C25	0.0312 (14)	0.0310 (15)	0.0361 (15)	−0.0044 (11)	−0.0020 (11)	−0.0015 (11)
C26	0.0311 (14)	0.0344 (15)	0.0351 (15)	−0.0019 (11)	−0.0018 (11)	−0.0017 (11)
C27	0.0486 (18)	0.0333 (16)	0.0457 (18)	−0.0035 (13)	−0.0157 (14)	0.0023 (13)
C28	0.0484 (18)	0.0381 (17)	0.0498 (19)	−0.0062 (13)	−0.0203 (15)	−0.0001 (14)
C29	0.0378 (15)	0.0446 (18)	0.0387 (16)	−0.0015 (13)	−0.0074 (13)	−0.0033 (13)
C30	0.0318 (14)	0.0356 (15)	0.0362 (15)	−0.0013 (11)	−0.0011 (11)	0.0022 (12)
C31	0.0245 (12)	0.0289 (13)	0.0324 (14)	−0.0019 (10)	−0.0043 (10)	−0.0049 (11)
C32	0.0270 (13)	0.0332 (14)	0.0332 (14)	−0.0033 (10)	−0.0034 (11)	−0.0024 (11)
C33	0.0259 (13)	0.0383 (15)	0.0358 (15)	−0.0031 (11)	−0.0015 (11)	−0.0054 (12)
C34	0.0269 (13)	0.0315 (14)	0.0417 (16)	0.0013 (10)	−0.0054 (11)	−0.0059 (12)
C35	0.0364 (15)	0.0340 (15)	0.0346 (15)	0.0016 (11)	−0.0071 (12)	−0.0009 (12)
C36	0.0309 (13)	0.0331 (14)	0.0338 (14)	−0.0009 (11)	−0.0010 (11)	−0.0015 (11)
O3	0.0405 (11)	0.0353 (11)	0.0348 (11)	−0.0049 (8)	−0.0015 (9)	−0.0060 (9)
O4	0.0720 (16)	0.0329 (12)	0.0419 (13)	0.0022 (11)	0.0008 (11)	−0.0011 (9)
N5	0.0280 (11)	0.0351 (12)	0.0298 (12)	−0.0009 (9)	−0.0043 (9)	−0.0001 (9)
N6	0.0288 (11)	0.0323 (12)	0.0315 (12)	−0.0001 (9)	−0.0046 (9)	−0.0027 (9)
N7	0.0281 (11)	0.0352 (13)	0.0344 (12)	−0.0026 (9)	−0.0043 (9)	−0.0018 (10)
N8	0.0299 (11)	0.0306 (12)	0.0312 (12)	−0.0026 (9)	−0.0040 (9)	−0.0006 (9)
N13	0.0357 (12)	0.0315 (12)	0.0321 (12)	−0.0051 (9)	0.0001 (10)	−0.0034 (10)
N14	0.0454 (15)	0.0549 (17)	0.0369 (14)	−0.0114 (12)	0.0014 (11)	−0.0103 (12)
N15	0.0367 (12)	0.0325 (12)	0.0324 (12)	0.0013 (10)	0.0002 (10)	0.0035 (10)
N16	0.0448 (14)	0.0405 (14)	0.0399 (14)	−0.0062 (11)	0.0002 (11)	0.0013 (11)
C5M	0.0292 (13)	0.0314 (14)	0.0315 (14)	−0.0022 (10)	−0.0027 (11)	0.0013 (11)
C6M	0.0335 (14)	0.0335 (14)	0.0265 (13)	−0.0002 (11)	−0.0007 (11)	−0.0043 (11)
C7M	0.0294 (13)	0.0329 (14)	0.0299 (14)	0.0003 (11)	0.0005 (11)	0.0013 (11)
C8M	0.0281 (13)	0.0334 (14)	0.0356 (15)	−0.0064 (11)	−0.0023 (11)	−0.0030 (11)
C9A	0.0278 (13)	0.0313 (14)	0.0313 (14)	−0.0016 (10)	−0.0017 (10)	0.0011 (11)
C9B	0.0320 (14)	0.0323 (14)	0.0322 (14)	−0.0001 (11)	0.0008 (11)	0.0003 (11)
C10A	0.0324 (14)	0.0320 (14)	0.0292 (13)	−0.0012 (11)	−0.0001 (11)	−0.0017 (11)
C10B	0.0359 (14)	0.0315 (14)	0.0290 (14)	−0.0011 (11)	0.0007 (11)	−0.0020 (11)
C11A	0.0312 (13)	0.0345 (15)	0.0315 (14)	−0.0017 (11)	−0.0025 (11)	−0.0029 (11)
C11B	0.0352 (15)	0.0356 (15)	0.0372 (15)	−0.0003 (12)	−0.0054 (12)	−0.0038 (12)
C12A	0.0303 (13)	0.0345 (15)	0.0320 (14)	0.0009 (11)	−0.0002 (11)	−0.0016 (11)
C12B	0.0333 (14)	0.0366 (15)	0.0370 (15)	−0.0007 (11)	−0.0068 (12)	−0.0025 (12)
C13A	0.0283 (13)	0.0320 (14)	0.0345 (14)	−0.0013 (10)	−0.0021 (11)	0.0006 (11)
C13B	0.0376 (15)	0.0365 (16)	0.0425 (17)	−0.0045 (12)	−0.0074 (13)	−0.0019 (13)
C14A	0.0289 (13)	0.0338 (15)	0.0365 (15)	−0.0031 (11)	−0.0019 (11)	−0.0032 (12)
C14B	0.0398 (16)	0.0387 (16)	0.0440 (17)	−0.0053 (12)	−0.0093 (13)	0.0000 (13)
C15A	0.0263 (13)	0.0327 (14)	0.0346 (14)	−0.0047 (10)	0.0001 (11)	−0.0004 (11)
C15B	0.0301 (13)	0.0379 (15)	0.0300 (14)	−0.0059 (11)	−0.0007 (11)	−0.0027 (11)
C16A	0.0269 (13)	0.0322 (14)	0.0319 (14)	−0.0031 (10)	−0.0009 (10)	0.0010 (11)
C16B	0.0298 (13)	0.0359 (15)	0.0317 (14)	−0.0034 (11)	−0.0044 (11)	−0.0008 (11)
C37	0.0344 (14)	0.0310 (14)	0.0319 (14)	−0.0019 (11)	−0.0054 (11)	−0.0037 (11)
C38	0.0372 (15)	0.0350 (16)	0.0504 (18)	−0.0047 (12)	−0.0080 (13)	0.0020 (13)
C39	0.0400 (16)	0.0430 (17)	0.0500 (19)	−0.0042 (13)	−0.0134 (14)	−0.0010 (14)
C40	0.0486 (18)	0.0396 (17)	0.0371 (16)	0.0056 (13)	−0.0149 (13)	−0.0027 (13)
C41	0.0493 (17)	0.0316 (15)	0.0315 (14)	−0.0029 (12)	−0.0056 (12)	0.0000 (11)
C42	0.0350 (14)	0.0308 (14)	0.0276 (13)	−0.0034 (11)	−0.0037 (11)	−0.0025 (11)
C43	0.0365 (14)	0.0334 (15)	0.0280 (13)	−0.0060 (11)	−0.0009 (11)	0.0000 (11)
C44	0.0370 (15)	0.0335 (15)	0.0302 (14)	−0.0069 (11)	0.0017 (11)	−0.0015 (11)
C45	0.0417 (16)	0.0435 (17)	0.0323 (15)	−0.0076 (13)	0.0010 (12)	−0.0033 (12)
C46	0.0395 (16)	0.059 (2)	0.0380 (17)	−0.0147 (14)	0.0062 (13)	−0.0078 (14)
C47	0.0405 (17)	0.056 (2)	0.0432 (18)	−0.0149 (14)	−0.0013 (14)	−0.0110 (15)
C48	0.0373 (15)	0.0491 (18)	0.0328 (15)	−0.0103 (13)	0.0008 (12)	−0.0053 (13)
C49	0.0339 (14)	0.0357 (15)	0.0316 (14)	0.0033 (11)	−0.0047 (11)	−0.0066 (11)
C50	0.0479 (17)	0.0403 (17)	0.0354 (16)	−0.0078 (13)	−0.0031 (13)	−0.0063 (13)
C51	0.055 (2)	0.0445 (18)	0.0455 (18)	−0.0083 (15)	−0.0079 (15)	−0.0074 (14)
C52	0.0499 (18)	0.0432 (18)	0.0457 (18)	0.0028 (14)	−0.0130 (14)	−0.0163 (14)
C53	0.0523 (19)	0.053 (2)	0.0312 (15)	0.0051 (15)	−0.0100 (13)	−0.0107 (14)
C54	0.0450 (17)	0.0402 (16)	0.0339 (15)	0.0030 (13)	−0.0068 (13)	−0.0050 (12)
C55	0.0298 (13)	0.0299 (14)	0.0342 (14)	−0.0034 (10)	−0.0040 (11)	−0.0010 (11)
C56	0.0291 (13)	0.0347 (15)	0.0347 (14)	−0.0062 (11)	−0.0009 (11)	−0.0001 (11)
C57	0.0378 (15)	0.0366 (15)	0.0313 (14)	−0.0097 (12)	−0.0042 (12)	0.0048 (12)
C58	0.0373 (15)	0.0345 (15)	0.0381 (16)	−0.0052 (12)	−0.0077 (12)	0.0070 (12)
C59	0.0340 (14)	0.0361 (15)	0.0361 (15)	−0.0002 (11)	−0.0017 (12)	0.0021 (12)
C60	0.0326 (14)	0.0300 (14)	0.0317 (14)	−0.0028 (11)	−0.0025 (11)	0.0020 (11)
C61	0.0395 (15)	0.0317 (15)	0.0361 (15)	−0.0047 (12)	0.0001 (12)	−0.0012 (12)
C62	0.0361 (15)	0.0324 (15)	0.0361 (15)	−0.0015 (11)	−0.0002 (12)	−0.0036 (12)
C63	0.0501 (18)	0.0339 (16)	0.0403 (17)	−0.0077 (13)	0.0021 (14)	−0.0067 (13)
C64	0.0560 (19)	0.0408 (17)	0.0346 (16)	−0.0008 (14)	−0.0007 (14)	−0.0063 (13)
C65	0.0545 (19)	0.0436 (18)	0.0319 (15)	−0.0005 (14)	0.0010 (13)	−0.0001 (13)
C66	0.0372 (15)	0.0388 (16)	0.0324 (15)	−0.0043 (12)	−0.0007 (12)	0.0000 (12)
C67	0.0367 (15)	0.0310 (14)	0.0364 (15)	−0.0043 (11)	−0.0057 (12)	−0.0006 (11)
C68	0.0369 (16)	0.0461 (18)	0.0482 (18)	−0.0095 (13)	−0.0019 (13)	−0.0086 (14)
C69	0.0451 (18)	0.053 (2)	0.062 (2)	−0.0161 (15)	−0.0107 (16)	−0.0114 (17)
C70	0.060 (2)	0.0418 (18)	0.056 (2)	−0.0073 (15)	−0.0137 (17)	−0.0151 (15)
C71	0.0485 (18)	0.0383 (17)	0.0502 (19)	−0.0011 (14)	−0.0026 (15)	−0.0111 (14)
C72	0.0417 (16)	0.0357 (16)	0.0448 (17)	−0.0030 (12)	−0.0042 (13)	−0.0054 (13)
C1S	0.052 (2)	0.049 (2)	0.059 (2)	−0.0066 (16)	0.0024 (16)	−0.0008 (16)
C2S	0.0486 (19)	0.051 (2)	0.060 (2)	−0.0106 (15)	0.0097 (16)	−0.0136 (17)
C3S	0.0378 (17)	0.053 (2)	0.067 (2)	−0.0040 (14)	−0.0002 (16)	−0.0136 (17)
C4S	0.0375 (16)	0.0445 (18)	0.056 (2)	0.0004 (13)	−0.0076 (14)	−0.0120 (15)
C5S	0.051 (2)	0.060 (2)	0.066 (2)	0.0106 (17)	−0.0164 (18)	−0.0052 (19)
C6S	0.062 (2)	0.047 (2)	0.065 (2)	0.0108 (17)	−0.0132 (19)	−0.0010 (17)
C73	0.056 (3)	0.064 (4)	0.094 (5)	0.017 (3)	−0.045 (3)	−0.012 (3)
C73A	0.078 (6)	0.112 (7)	0.129 (7)	0.028 (6)	−0.048 (6)	0.011 (7)
C74	0.100 (5)	0.091 (5)	0.146 (7)	−0.021 (4)	−0.056 (5)	0.004 (5)
C74A	0.046 (6)	0.153 (10)	0.055 (6)	0.065 (6)	−0.031 (5)	−0.054 (7)
C75	0.077 (5)	0.068 (4)	0.130 (6)	0.000 (3)	0.009 (4)	−0.001 (4)
C75A	0.095 (5)	0.086 (6)	0.143 (7)	0.002 (5)	−0.034 (6)	−0.008 (6)
C76	0.093 (5)	0.077 (4)	0.154 (7)	−0.014 (4)	0.039 (5)	−0.007 (5)
C76A	0.103 (6)	0.078 (5)	0.154 (8)	−0.008 (5)	0.007 (6)	−0.007 (6)
C77	0.114 (6)	0.058 (4)	0.159 (7)	0.000 (4)	0.044 (5)	−0.012 (5)
C77A	0.104 (6)	0.067 (5)	0.154 (8)	−0.017 (5)	0.029 (6)	−0.019 (6)
C78	0.172 (9)	0.103 (7)	0.194 (10)	−0.038 (7)	0.011 (8)	−0.013 (7)
C78A	0.149 (12)	0.073 (9)	0.182 (13)	−0.027 (9)	−0.008 (11)	−0.026 (10)

### Geometric parameters (Å, º)

**Table:**

O1—C7	1.221 (4)	C7M—C13A	1.400 (4)
O2—C25	1.218 (3)	C7M—C55	1.502 (4)
N1—C1A	1.369 (3)	C8M—C14A	1.405 (4)
N1—C2A	1.370 (3)	C8M—C15A	1.401 (4)
N2—H2	0.8800	C8M—C67	1.500 (4)
N2—C3A	1.377 (3)	C9A—C9B	1.444 (4)
N2—C4A	1.367 (3)	C9B—H9B	0.9500
N3—C5A	1.368 (3)	C9B—C10B	1.358 (4)
N3—C6A	1.369 (3)	C10A—C10B	1.446 (4)
N4—H4	0.8800	C10B—H10B	0.9500
N4—C7A	1.372 (3)	C11A—C11B	1.434 (4)
N4—C8A	1.374 (3)	C11B—H11B	0.9500
N9—H9	0.8800	C11B—C12B	1.369 (4)
N9—C6	1.418 (3)	C12A—C12B	1.437 (4)
N9—C7	1.355 (4)	C12B—H12B	0.9500
N10—C11	1.332 (4)	C13A—C13B	1.464 (4)
N10—C12	1.345 (4)	C13B—H13B	0.9500
N11—H11	0.8800	C13B—C14B	1.401 (4)
N11—C24	1.418 (3)	C14A—C14B	1.467 (4)
N11—C25	1.366 (4)	C14B—H14B	0.9500
N12—C29	1.330 (4)	C15A—C15B	1.434 (4)
N12—C30	1.341 (4)	C15B—H15B	0.9500
C1—C1M	1.494 (4)	C15B—C16B	1.356 (4)
C1—C2	1.388 (4)	C16A—C16B	1.431 (4)
C1—C6	1.406 (4)	C16B—H16B	0.9500
C1A—C1B	1.448 (4)	C37—C38	1.392 (4)
C1A—C1M	1.405 (4)	C37—C42	1.408 (4)
C1B—H1B	0.9500	C38—H38	0.9500
C1B—C2B	1.357 (4)	C38—C39	1.390 (4)
C1M—C8A	1.396 (4)	C39—H39	0.9500
C2—H2A	0.9500	C39—C40	1.387 (5)
C2—C3	1.389 (4)	C40—H40	0.9500
C2A—C2B	1.447 (4)	C40—C41	1.378 (4)
C2A—C2M	1.415 (4)	C41—H41	0.9500
C2B—H2B	0.9500	C41—C42	1.392 (4)
C2M—C3A	1.397 (4)	C43—C44	1.501 (4)
C2M—C13	1.486 (4)	C44—C45	1.382 (4)
C3—H3	0.9500	C44—C48	1.392 (4)
C3—C4	1.373 (4)	C45—H45	0.9500
C3A—C3B	1.438 (4)	C45—C46	1.388 (4)
C3B—H3B	0.9500	C46—H46	0.9500
C3B—C4B	1.365 (4)	C46—C47	1.379 (5)
C3M—C4A	1.387 (4)	C47—H47	0.9500
C3M—C5A	1.412 (4)	C48—H48	0.9500
C3M—C19	1.494 (4)	C49—C50	1.391 (4)
C4—H4A	0.9500	C49—C54	1.393 (4)
C4—C5	1.390 (4)	C50—H50	0.9500
C4A—C4B	1.440 (4)	C50—C51	1.390 (4)
C4B—H4B	0.9500	C51—H51	0.9500
C4M—C6A	1.412 (4)	C51—C52	1.385 (5)
C4M—C7A	1.397 (4)	C52—H52	0.9500
C4M—C31	1.485 (3)	C52—C53	1.376 (5)
C5—H5	0.9500	C53—H53	0.9500
C5—C6	1.391 (4)	C53—C54	1.395 (4)
C5A—C5B	1.451 (4)	C54—H54	0.9500
C5B—H5B	0.9500	C55—C56	1.391 (4)
C5B—C6B	1.367 (4)	C55—C60	1.402 (4)
C6A—C6B	1.456 (4)	C56—H56	0.9500
C6B—H6B	0.9500	C56—C57	1.384 (4)
C7—C8	1.499 (4)	C57—H57	0.9500
C7A—C7B	1.432 (4)	C57—C58	1.386 (4)
C7B—H7B	0.9500	C58—H58	0.9500
C7B—C8B	1.366 (4)	C58—C59	1.388 (4)
C8—C9	1.389 (4)	C59—H59	0.9500
C8—C12	1.389 (4)	C59—C60	1.394 (4)
C8A—C8B	1.434 (4)	C61—C62	1.497 (4)
C8B—H8B	0.9500	C62—C63	1.382 (4)
C9—H9A	0.9500	C62—C66	1.397 (4)
C9—C10	1.383 (5)	C63—H63	0.9500
C10—H10	0.9500	C63—C64	1.385 (4)
C10—C11	1.384 (5)	C64—H64	0.9500
C11—H11A	0.9500	C64—C65	1.372 (5)
C12—H12	0.9500	C65—H65	0.9500
C13—C14	1.402 (4)	C66—H66	0.9500
C13—C18	1.395 (4)	C67—C68	1.391 (4)
C14—H14	0.9500	C67—C72	1.395 (4)
C14—C15	1.396 (4)	C68—H68	0.9500
C15—H15	0.9500	C68—C69	1.392 (5)
C15—C16	1.378 (5)	C69—H69	0.9500
C16—H16	0.9500	C69—C70	1.377 (5)
C16—C17	1.379 (4)	C70—H70	0.9500
C17—H17	0.9500	C70—C71	1.387 (5)
C17—C18	1.389 (4)	C71—H71	0.9500
C18—H18	0.9500	C71—C72	1.391 (4)
C19—C20	1.399 (4)	C72—H72	0.9500
C19—C24	1.398 (4)	C1S—H1SA	0.9800
C20—H20	0.9500	C1S—H1SB	0.9800
C20—C21	1.387 (4)	C1S—H1SC	0.9800
C21—H21	0.9500	C1S—C2S	1.501 (5)
C21—C22	1.385 (5)	C2S—H2SA	0.9900
C22—H22	0.9500	C2S—H2SB	0.9900
C22—C23	1.383 (4)	C2S—C3S	1.538 (5)
C23—H23	0.9500	C3S—H3SA	0.9900
C23—C24	1.401 (4)	C3S—H3SB	0.9900
C25—C26	1.503 (4)	C3S—C4S	1.530 (5)
C26—C27	1.380 (4)	C4S—H4SA	0.9900
C26—C30	1.388 (4)	C4S—H4SB	0.9900
C27—H27	0.9500	C4S—C5S	1.516 (5)
C27—C28	1.387 (4)	C5S—H5SA	0.9900
C28—H28	0.9500	C5S—H5SB	0.9900
C28—C29	1.383 (4)	C5S—C6S	1.506 (6)
C29—H29	0.9500	C6S—H6SA	0.9800
C30—H30	0.9500	C6S—H6SB	0.9800
C31—C32	1.396 (4)	C6S—H6SC	0.9800
C31—C36	1.404 (4)	C73—H73A	0.9800
C32—H32	0.9500	C73—H73B	0.9800
C32—C33	1.390 (4)	C73—H73C	0.9800
C33—H33	0.9500	C73—C74	1.376 (12)
C33—C34	1.383 (4)	C73A—H73D	0.9900
C34—H34	0.9500	C73A—H73E	0.9900
C34—C35	1.393 (4)	C73A—C74A	1.564 (16)
C35—H35	0.9500	C73A—C75A	1.690 (18)
C35—C36	1.381 (4)	C74—H74A	0.9900
C36—H36	0.9500	C74—H74B	0.9900
O3—C43	1.235 (3)	C74—C75	1.564 (16)
O4—C61	1.221 (4)	C74A—H74C	0.9800
N5—C9A	1.367 (3)	C74A—H74D	0.9800
N5—C10A	1.376 (4)	C74A—H74E	0.9800
N6—H6	0.8800	C75—H75A	0.9900
N6—C11A	1.367 (4)	C75—H75B	0.9900
N6—C12A	1.365 (3)	C75—C76	1.559 (14)
N7—C13A	1.366 (3)	C75A—H75C	0.9900
N7—C14A	1.367 (4)	C75A—H75D	0.9900
N8—H8	0.8800	C75A—C76A	1.545 (18)
N8—C15A	1.375 (4)	C76—H76A	0.9900
N8—C16A	1.373 (3)	C76—H76B	0.9900
N13—H13	0.8800	C76—C77	1.718 (18)
N13—C42	1.421 (4)	C76A—H76C	0.9900
N13—C43	1.350 (4)	C76A—H76D	0.9900
N14—C47	1.340 (4)	C76A—C77A	1.615 (17)
N14—C48	1.333 (4)	C77—H77A	0.9900
N15—H15A	0.8800	C77—H77B	0.9900
N15—C60	1.414 (4)	C77—C78	1.372 (13)
N15—C61	1.368 (4)	C77A—H77C	0.9900
N16—C65	1.345 (4)	C77A—H77D	0.9900
N16—C66	1.340 (4)	C77A—C78A	1.581 (17)
C5M—C9A	1.410 (4)	C78—H78A	0.9800
C5M—C16A	1.404 (4)	C78—H78B	0.9800
C5M—C37	1.494 (4)	C78—H78C	0.9800
C6M—C10A	1.405 (4)	C78A—H78D	0.9800
C6M—C11A	1.400 (4)	C78A—H78E	0.9800
C6M—C49	1.499 (4)	C78A—H78F	0.9800
C7M—C12A	1.399 (4)		
			
C1A—N1—C2A	105.2 (2)	C7M—C13A—C13B	123.9 (3)
C3A—N2—H2	124.9	C13A—C13B—H13B	126.9
C4A—N2—H2	124.9	C14B—C13B—C13A	106.1 (3)
C4A—N2—C3A	110.3 (2)	C14B—C13B—H13B	126.9
C5A—N3—C6A	105.6 (2)	N7—C14A—C8M	125.4 (3)
C7A—N4—H4	125.0	N7—C14A—C14B	110.6 (2)
C7A—N4—C8A	110.1 (2)	C8M—C14A—C14B	123.8 (3)
C8A—N4—H4	125.0	C13B—C14B—C14A	105.5 (3)
C6—N9—H9	116.2	C13B—C14B—H14B	127.3
C7—N9—H9	116.2	C14A—C14B—H14B	127.3
C7—N9—C6	127.6 (2)	N8—C15A—C8M	126.3 (3)
C11—N10—C12	117.6 (3)	N8—C15A—C15B	107.2 (2)
C24—N11—H11	118.4	C8M—C15A—C15B	126.5 (3)
C25—N11—H11	118.4	C15A—C15B—H15B	126.0
C25—N11—C24	123.3 (2)	C16B—C15B—C15A	108.1 (3)
C29—N12—C30	117.8 (3)	C16B—C15B—H15B	126.0
C2—C1—C1M	121.0 (2)	N8—C16A—C5M	125.9 (3)
C2—C1—C6	118.8 (2)	N8—C16A—C16B	107.5 (2)
C6—C1—C1M	120.2 (2)	C5M—C16A—C16B	126.5 (3)
N1—C1A—C1B	111.0 (2)	C15B—C16B—C16A	107.9 (2)
N1—C1A—C1M	126.0 (2)	C15B—C16B—H16B	126.1
C1M—C1A—C1B	122.8 (2)	C16A—C16B—H16B	126.1
C1A—C1B—H1B	126.9	C38—C37—C5M	119.9 (3)
C2B—C1B—C1A	106.3 (2)	C38—C37—C42	117.5 (3)
C2B—C1B—H1B	126.9	C42—C37—C5M	122.6 (2)
C1A—C1M—C1	117.0 (2)	C37—C38—H38	118.9
C8A—C1M—C1	116.5 (2)	C39—C38—C37	122.3 (3)
C8A—C1M—C1A	126.4 (2)	C39—C38—H38	118.9
C1—C2—H2A	119.5	C38—C39—H39	120.4
C1—C2—C3	121.0 (3)	C40—C39—C38	119.2 (3)
C3—C2—H2A	119.5	C40—C39—H39	120.4
N1—C2A—C2B	110.8 (2)	C39—C40—H40	120.1
N1—C2A—C2M	125.2 (2)	C41—C40—C39	119.9 (3)
C2M—C2A—C2B	123.9 (2)	C41—C40—H40	120.1
C1B—C2B—C2A	106.6 (2)	C40—C41—H41	119.5
C1B—C2B—H2B	126.7	C40—C41—C42	120.9 (3)
C2A—C2B—H2B	126.7	C42—C41—H41	119.5
C2A—C2M—C13	118.0 (2)	C37—C42—N13	122.2 (2)
C3A—C2M—C2A	124.3 (2)	C41—C42—N13	117.5 (2)
C3A—C2M—C13	117.6 (2)	C41—C42—C37	120.2 (3)
C2—C3—H3	120.2	O3—C43—N13	123.9 (3)
C4—C3—C2	119.5 (3)	O3—C43—C44	122.7 (3)
C4—C3—H3	120.2	N13—C43—C44	113.4 (2)
N2—C3A—C2M	125.4 (2)	C45—C44—C43	121.8 (3)
N2—C3A—C3B	106.8 (2)	C45—C44—C48	118.2 (3)
C2M—C3A—C3B	127.8 (2)	C48—C44—C43	120.0 (2)
C3A—C3B—H3B	126.0	C44—C45—H45	120.9
C4B—C3B—C3A	108.1 (2)	C44—C45—C46	118.2 (3)
C4B—C3B—H3B	126.0	C46—C45—H45	120.9
C4A—C3M—C5A	126.4 (2)	C45—C46—H46	120.4
C4A—C3M—C19	116.2 (2)	C47—C46—C45	119.2 (3)
C5A—C3M—C19	117.3 (2)	C47—C46—H46	120.4
C3—C4—H4A	119.5	N14—C47—C46	123.6 (3)
C3—C4—C5	121.0 (3)	N14—C47—H47	118.2
C5—C4—H4A	119.5	C46—C47—H47	118.2
N2—C4A—C3M	127.5 (2)	N14—C48—C44	124.4 (3)
N2—C4A—C4B	107.1 (2)	N14—C48—H48	117.8
C3M—C4A—C4B	125.4 (2)	C44—C48—H48	117.8
C3B—C4B—C4A	107.8 (2)	C50—C49—C6M	120.4 (3)
C3B—C4B—H4B	126.1	C50—C49—C54	118.9 (3)
C4A—C4B—H4B	126.1	C54—C49—C6M	120.7 (3)
C6A—C4M—C31	118.4 (2)	C49—C50—H50	119.8
C7A—C4M—C6A	124.4 (2)	C51—C50—C49	120.4 (3)
C7A—C4M—C31	117.2 (2)	C51—C50—H50	119.8
C4—C5—H5	120.2	C50—C51—H51	119.8
C4—C5—C6	119.5 (3)	C52—C51—C50	120.4 (3)
C6—C5—H5	120.2	C52—C51—H51	119.8
N3—C5A—C3M	125.8 (2)	C51—C52—H52	120.2
N3—C5A—C5B	110.9 (2)	C53—C52—C51	119.5 (3)
C3M—C5A—C5B	123.2 (2)	C53—C52—H52	120.2
C5A—C5B—H5B	126.8	C52—C53—H53	119.7
C6B—C5B—C5A	106.4 (2)	C52—C53—C54	120.5 (3)
C6B—C5B—H5B	126.8	C54—C53—H53	119.7
C1—C6—N9	118.0 (2)	C49—C54—C53	120.3 (3)
C5—C6—N9	121.8 (3)	C49—C54—H54	119.9
C5—C6—C1	120.1 (2)	C53—C54—H54	119.9
N3—C6A—C4M	125.3 (2)	C56—C55—C7M	119.9 (2)
N3—C6A—C6B	110.8 (2)	C56—C55—C60	119.1 (3)
C4M—C6A—C6B	123.9 (2)	C60—C55—C7M	121.0 (2)
C5B—C6B—C6A	106.1 (2)	C55—C56—H56	119.2
C5B—C6B—H6B	126.9	C57—C56—C55	121.6 (3)
C6A—C6B—H6B	126.9	C57—C56—H56	119.2
O1—C7—N9	123.9 (3)	C56—C57—H57	120.5
O1—C7—C8	120.0 (3)	C56—C57—C58	118.9 (3)
N9—C7—C8	116.1 (2)	C58—C57—H57	120.5
N4—C7A—C4M	126.1 (2)	C57—C58—H58	119.7
N4—C7A—C7B	107.0 (2)	C57—C58—C59	120.6 (3)
C4M—C7A—C7B	126.9 (2)	C59—C58—H58	119.7
C7A—C7B—H7B	126.0	C58—C59—H59	119.8
C8B—C7B—C7A	108.1 (2)	C58—C59—C60	120.4 (3)
C8B—C7B—H7B	126.0	C60—C59—H59	119.8
C9—C8—C7	117.2 (3)	C55—C60—N15	119.3 (2)
C9—C8—C12	118.3 (3)	C59—C60—N15	121.3 (2)
C12—C8—C7	124.4 (3)	C59—C60—C55	119.3 (3)
N4—C8A—C1M	126.7 (2)	O4—C61—N15	124.6 (3)
N4—C8A—C8B	107.0 (2)	O4—C61—C62	120.9 (3)
C1M—C8A—C8B	126.3 (2)	N15—C61—C62	114.5 (2)
C7B—C8B—C8A	107.9 (2)	C63—C62—C61	120.1 (3)
C7B—C8B—H8B	126.1	C63—C62—C66	118.4 (3)
C8A—C8B—H8B	126.1	C66—C62—C61	121.5 (3)
C8—C9—H9A	120.5	C62—C63—H63	120.4
C10—C9—C8	119.0 (3)	C62—C63—C64	119.1 (3)
C10—C9—H9A	120.5	C64—C63—H63	120.4
C9—C10—H10	120.7	C63—C64—H64	120.9
C9—C10—C11	118.5 (3)	C65—C64—C63	118.1 (3)
C11—C10—H10	120.7	C65—C64—H64	120.9
N10—C11—C10	123.5 (3)	N16—C65—C64	124.6 (3)
N10—C11—H11A	118.2	N16—C65—H65	117.7
C10—C11—H11A	118.2	C64—C65—H65	117.7
N10—C12—C8	123.0 (3)	N16—C66—C62	123.3 (3)
N10—C12—H12	118.5	N16—C66—H66	118.4
C8—C12—H12	118.5	C62—C66—H66	118.4
C14—C13—C2M	121.0 (3)	C68—C67—C8M	121.4 (3)
C18—C13—C2M	119.9 (2)	C68—C67—C72	118.9 (3)
C18—C13—C14	119.0 (3)	C72—C67—C8M	119.6 (3)
C13—C14—H14	120.1	C67—C68—H68	119.9
C15—C14—C13	119.9 (3)	C67—C68—C69	120.3 (3)
C15—C14—H14	120.1	C69—C68—H68	119.9
C14—C15—H15	120.0	C68—C69—H69	119.8
C16—C15—C14	120.0 (3)	C70—C69—C68	120.5 (3)
C16—C15—H15	120.0	C70—C69—H69	119.8
C15—C16—H16	119.7	C69—C70—H70	120.0
C15—C16—C17	120.6 (3)	C69—C70—C71	119.9 (3)
C17—C16—H16	119.7	C71—C70—H70	120.0
C16—C17—H17	120.0	C70—C71—H71	120.1
C16—C17—C18	120.0 (3)	C70—C71—C72	119.9 (3)
C18—C17—H17	120.0	C72—C71—H71	120.1
C13—C18—H18	119.8	C67—C72—H72	119.7
C17—C18—C13	120.4 (3)	C71—C72—C67	120.5 (3)
C17—C18—H18	119.8	C71—C72—H72	119.7
C20—C19—C3M	120.5 (2)	H1SA—C1S—H1SB	109.5
C24—C19—C3M	120.7 (2)	H1SA—C1S—H1SC	109.5
C24—C19—C20	118.8 (2)	H1SB—C1S—H1SC	109.5
C19—C20—H20	119.5	C2S—C1S—H1SA	109.5
C21—C20—C19	121.0 (3)	C2S—C1S—H1SB	109.5
C21—C20—H20	119.5	C2S—C1S—H1SC	109.5
C20—C21—H21	120.3	C1S—C2S—H2SA	108.7
C22—C21—C20	119.5 (3)	C1S—C2S—H2SB	108.7
C22—C21—H21	120.3	C1S—C2S—C3S	114.2 (3)
C21—C22—H22	119.6	H2SA—C2S—H2SB	107.6
C23—C22—C21	120.8 (3)	C3S—C2S—H2SA	108.7
C23—C22—H22	119.6	C3S—C2S—H2SB	108.7
C22—C23—H23	120.1	C2S—C3S—H3SA	108.9
C22—C23—C24	119.9 (3)	C2S—C3S—H3SB	108.9
C24—C23—H23	120.1	H3SA—C3S—H3SB	107.7
C19—C24—N11	118.7 (2)	C4S—C3S—C2S	113.4 (3)
C19—C24—C23	120.0 (3)	C4S—C3S—H3SA	108.9
C23—C24—N11	121.3 (3)	C4S—C3S—H3SB	108.9
O2—C25—N11	123.5 (3)	C3S—C4S—H4SA	108.7
O2—C25—C26	121.4 (3)	C3S—C4S—H4SB	108.7
N11—C25—C26	115.1 (2)	H4SA—C4S—H4SB	107.6
C27—C26—C25	124.3 (3)	C5S—C4S—C3S	114.3 (3)
C27—C26—C30	118.1 (3)	C5S—C4S—H4SA	108.7
C30—C26—C25	117.5 (3)	C5S—C4S—H4SB	108.7
C26—C27—H27	120.6	C4S—C5S—H5SA	108.9
C26—C27—C28	118.8 (3)	C4S—C5S—H5SB	108.9
C28—C27—H27	120.6	H5SA—C5S—H5SB	107.7
C27—C28—H28	120.5	C6S—C5S—C4S	113.4 (3)
C29—C28—C27	119.1 (3)	C6S—C5S—H5SA	108.9
C29—C28—H28	120.5	C6S—C5S—H5SB	108.9
N12—C29—C28	122.7 (3)	C5S—C6S—H6SA	109.5
N12—C29—H29	118.6	C5S—C6S—H6SB	109.5
C28—C29—H29	118.6	C5S—C6S—H6SC	109.5
N12—C30—C26	123.3 (3)	H6SA—C6S—H6SB	109.5
N12—C30—H30	118.3	H6SA—C6S—H6SC	109.5
C26—C30—H30	118.3	H6SB—C6S—H6SC	109.5
C32—C31—C4M	120.3 (2)	H73A—C73—H73B	109.5
C32—C31—C36	118.5 (2)	H73A—C73—H73C	109.5
C36—C31—C4M	121.3 (2)	H73B—C73—H73C	109.5
C31—C32—H32	119.7	C74—C73—H73A	109.5
C33—C32—C31	120.6 (3)	C74—C73—H73B	109.5
C33—C32—H32	119.7	C74—C73—H73C	109.5
C32—C33—H33	119.9	H73D—C73A—H73E	105.2
C34—C33—C32	120.2 (3)	C74A—C73A—H73D	103.2
C34—C33—H33	119.9	C74A—C73A—H73E	103.2
C33—C34—H34	120.0	C74A—C73A—C75A	135.7 (18)
C33—C34—C35	120.0 (3)	C75A—C73A—H73D	103.2
C35—C34—H34	120.0	C75A—C73A—H73E	103.2
C34—C35—H35	120.0	C73—C74—H74A	108.1
C36—C35—C34	119.9 (3)	C73—C74—H74B	108.1
C36—C35—H35	120.0	C73—C74—C75	117.0 (10)
C31—C36—H36	119.6	H74A—C74—H74B	107.3
C35—C36—C31	120.8 (3)	C75—C74—H74A	108.1
C35—C36—H36	119.6	C75—C74—H74B	108.1
C9A—N5—C10A	106.2 (2)	C73A—C74A—H74C	109.5
C11A—N6—H6	125.1	C73A—C74A—H74D	109.5
C12A—N6—H6	125.1	C73A—C74A—H74E	109.5
C12A—N6—C11A	109.8 (2)	H74C—C74A—H74D	109.5
C13A—N7—C14A	107.1 (2)	H74C—C74A—H74E	109.5
C15A—N8—H8	125.4	H74D—C74A—H74E	109.5
C16A—N8—H8	125.4	C74—C75—H75A	108.1
C16A—N8—C15A	109.3 (2)	C74—C75—H75B	108.1
C42—N13—H13	117.4	H75A—C75—H75B	107.3
C43—N13—H13	117.4	C76—C75—C74	117.0 (10)
C43—N13—C42	125.3 (2)	C76—C75—H75A	108.1
C48—N14—C47	116.4 (3)	C76—C75—H75B	108.1
C60—N15—H15A	117.4	C73A—C75A—H75C	105.2
C61—N15—H15A	117.4	C73A—C75A—H75D	105.2
C61—N15—C60	125.1 (2)	H75C—C75A—H75D	105.9
C66—N16—C65	116.4 (3)	C76A—C75A—C73A	128 (2)
C9A—C5M—C37	117.5 (2)	C76A—C75A—H75C	105.2
C16A—C5M—C9A	125.3 (2)	C76A—C75A—H75D	105.2
C16A—C5M—C37	117.2 (2)	C75—C76—H76A	109.8
C10A—C6M—C49	117.5 (2)	C75—C76—H76B	109.8
C11A—C6M—C10A	124.7 (3)	C75—C76—C77	109.4 (9)
C11A—C6M—C49	117.8 (2)	H76A—C76—H76B	108.2
C12A—C7M—C13A	126.0 (3)	C77—C76—H76A	109.8
C12A—C7M—C55	116.7 (2)	C77—C76—H76B	109.8
C13A—C7M—C55	117.3 (2)	C75A—C76A—H76C	102.3
C14A—C8M—C67	117.8 (3)	C75A—C76A—H76D	102.3
C15A—C8M—C14A	125.2 (3)	C75A—C76A—C77A	139 (2)
C15A—C8M—C67	116.9 (2)	H76C—C76A—H76D	104.9
N5—C9A—C5M	125.4 (2)	C77A—C76A—H76C	102.3
N5—C9A—C9B	110.2 (2)	C77A—C76A—H76D	102.3
C5M—C9A—C9B	124.4 (2)	C76—C77—H77A	111.3
C9A—C9B—H9B	126.5	C76—C77—H77B	111.3
C10B—C9B—C9A	106.9 (2)	H77A—C77—H77B	109.2
C10B—C9B—H9B	126.5	C78—C77—C76	102.3 (11)
N5—C10A—C6M	125.4 (2)	C78—C77—H77A	111.3
N5—C10A—C10B	109.9 (2)	C78—C77—H77B	111.3
C6M—C10A—C10B	124.6 (3)	C76A—C77A—H77C	101.9
C9B—C10B—C10A	106.7 (2)	C76A—C77A—H77D	101.9
C9B—C10B—H10B	126.6	H77C—C77A—H77D	104.7
C10A—C10B—H10B	126.6	C78A—C77A—C76A	140 (2)
N6—C11A—C6M	126.1 (3)	C78A—C77A—H77C	101.9
N6—C11A—C11B	107.8 (2)	C78A—C77A—H77D	101.9
C6M—C11A—C11B	126.1 (3)	C77—C78—H78A	109.5
C11A—C11B—H11B	126.4	C77—C78—H78B	109.5
C12B—C11B—C11A	107.2 (3)	C77—C78—H78C	109.5
C12B—C11B—H11B	126.4	H78A—C78—H78B	109.5
N6—C12A—C7M	126.6 (3)	H78A—C78—H78C	109.5
N6—C12A—C12B	107.3 (2)	H78B—C78—H78C	109.5
C7M—C12A—C12B	126.1 (3)	C77A—C78A—H78D	109.5
C11B—C12B—C12A	107.9 (3)	C77A—C78A—H78E	109.5
C11B—C12B—H12B	126.1	C77A—C78A—H78F	109.5
C12A—C12B—H12B	126.1	H78D—C78A—H78E	109.5
N7—C13A—C7M	125.6 (3)	H78D—C78A—H78F	109.5
N7—C13A—C13B	110.4 (2)	H78E—C78A—H78F	109.5
			
O1—C7—C8—C9	31.0 (5)	N5—C10A—C10B—C9B	1.8 (3)
O1—C7—C8—C12	−146.2 (3)	N6—C11A—C11B—C12B	−0.2 (3)
O2—C25—C26—C27	−150.1 (3)	N6—C12A—C12B—C11B	−1.6 (3)
O2—C25—C26—C30	26.6 (4)	N7—C13A—C13B—C14B	−3.0 (3)
N1—C1A—C1B—C2B	−1.6 (3)	N7—C14A—C14B—C13B	2.9 (3)
N1—C1A—C1M—C1	169.0 (2)	N8—C15A—C15B—C16B	0.2 (3)
N1—C1A—C1M—C8A	−7.6 (4)	N8—C16A—C16B—C15B	−1.4 (3)
N1—C2A—C2B—C1B	1.6 (3)	N13—C43—C44—C45	132.9 (3)
N1—C2A—C2M—C3A	8.1 (4)	N13—C43—C44—C48	−48.7 (4)
N1—C2A—C2M—C13	−169.9 (2)	N15—C61—C62—C63	127.8 (3)
N2—C3A—C3B—C4B	0.2 (3)	N15—C61—C62—C66	−52.0 (4)
N2—C4A—C4B—C3B	−1.2 (3)	C5M—C9A—C9B—C10B	175.4 (3)
N3—C5A—C5B—C6B	−3.5 (3)	C5M—C16A—C16B—C15B	177.0 (3)
N3—C6A—C6B—C5B	−0.8 (3)	C5M—C37—C38—C39	−178.2 (3)
N4—C7A—C7B—C8B	−0.7 (3)	C5M—C37—C42—N13	1.2 (4)
N4—C8A—C8B—C7B	0.1 (3)	C5M—C37—C42—C41	177.6 (3)
N9—C7—C8—C9	−150.7 (3)	C6M—C10A—C10B—C9B	−176.3 (3)
N9—C7—C8—C12	32.1 (4)	C6M—C11A—C11B—C12B	179.3 (3)
N11—C25—C26—C27	30.2 (4)	C6M—C49—C50—C51	−177.4 (3)
N11—C25—C26—C30	−153.1 (3)	C6M—C49—C54—C53	177.7 (3)
C1—C1M—C8A—N4	178.7 (2)	C7M—C12A—C12B—C11B	178.2 (3)
C1—C1M—C8A—C8B	−3.4 (4)	C7M—C13A—C13B—C14B	172.6 (3)
C1—C2—C3—C4	−1.8 (4)	C7M—C55—C56—C57	−178.5 (3)
C1A—N1—C2A—C2B	−2.5 (3)	C7M—C55—C60—N15	−4.2 (4)
C1A—N1—C2A—C2M	176.3 (3)	C7M—C55—C60—C59	178.0 (3)
C1A—C1B—C2B—C2A	0.0 (3)	C8M—C14A—C14B—C13B	−172.6 (3)
C1A—C1M—C8A—N4	−4.7 (4)	C8M—C15A—C15B—C16B	−178.9 (3)
C1A—C1M—C8A—C8B	173.2 (3)	C8M—C67—C68—C69	−177.0 (3)
C1B—C1A—C1M—C1	−6.4 (4)	C8M—C67—C72—C71	177.5 (3)
C1B—C1A—C1M—C8A	177.1 (3)	C9A—N5—C10A—C6M	175.0 (3)
C1M—C1—C2—C3	−177.7 (2)	C9A—N5—C10A—C10B	−3.0 (3)
C1M—C1—C6—N9	−1.3 (4)	C9A—C5M—C16A—N8	−4.2 (4)
C1M—C1—C6—C5	179.5 (2)	C9A—C5M—C16A—C16B	177.6 (3)
C1M—C1A—C1B—C2B	174.4 (3)	C9A—C5M—C37—C38	117.0 (3)
C1M—C8A—C8B—C7B	−178.1 (3)	C9A—C5M—C37—C42	−61.8 (4)
C2—C1—C1M—C1A	111.1 (3)	C9A—C9B—C10B—C10A	0.2 (3)
C2—C1—C1M—C8A	−72.0 (3)	C10A—N5—C9A—C5M	−174.3 (3)
C2—C1—C6—N9	179.3 (2)	C10A—N5—C9A—C9B	3.2 (3)
C2—C1—C6—C5	0.1 (4)	C10A—C6M—C11A—N6	9.4 (5)
C2—C3—C4—C5	0.3 (4)	C10A—C6M—C11A—C11B	−170.0 (3)
C2A—N1—C1A—C1B	2.5 (3)	C10A—C6M—C49—C50	59.3 (4)
C2A—N1—C1A—C1M	−173.3 (3)	C10A—C6M—C49—C54	−119.1 (3)
C2A—C2M—C3A—N2	1.1 (4)	C11A—N6—C12A—C7M	−178.3 (3)
C2A—C2M—C3A—C3B	179.3 (3)	C11A—N6—C12A—C12B	1.5 (3)
C2A—C2M—C13—C14	−122.7 (3)	C11A—C6M—C10A—N5	7.8 (4)
C2A—C2M—C13—C18	60.1 (3)	C11A—C6M—C10A—C10B	−174.5 (3)
C2B—C2A—C2M—C3A	−173.2 (3)	C11A—C6M—C49—C50	−119.9 (3)
C2B—C2A—C2M—C13	8.7 (4)	C11A—C6M—C49—C54	61.7 (4)
C2M—C2A—C2B—C1B	−177.2 (3)	C11A—C11B—C12B—C12A	1.1 (3)
C2M—C3A—C3B—C4B	−178.2 (3)	C12A—N6—C11A—C6M	179.6 (3)
C2M—C13—C14—C15	−177.1 (3)	C12A—N6—C11A—C11B	−0.8 (3)
C2M—C13—C18—C17	175.6 (3)	C12A—C7M—C13A—N7	−9.1 (5)
C3—C4—C5—C6	1.4 (4)	C12A—C7M—C13A—C13B	176.0 (3)
C3A—N2—C4A—C3M	179.7 (3)	C12A—C7M—C55—C56	−75.9 (3)
C3A—N2—C4A—C4B	1.3 (3)	C12A—C7M—C55—C60	103.7 (3)
C3A—C2M—C13—C14	59.1 (4)	C13A—N7—C14A—C8M	170.6 (3)
C3A—C2M—C13—C18	−118.1 (3)	C13A—N7—C14A—C14B	−4.7 (3)
C3A—C3B—C4B—C4A	0.6 (3)	C13A—C7M—C12A—N6	−5.6 (5)
C3M—C4A—C4B—C3B	−179.6 (3)	C13A—C7M—C12A—C12B	174.6 (3)
C3M—C5A—C5B—C6B	173.5 (2)	C13A—C7M—C55—C56	105.5 (3)
C3M—C19—C20—C21	−178.5 (2)	C13A—C7M—C55—C60	−74.9 (3)
C3M—C19—C24—N11	−3.3 (4)	C13A—C13B—C14B—C14A	0.1 (3)
C3M—C19—C24—C23	177.5 (2)	C14A—N7—C13A—C7M	−170.7 (3)
C4—C5—C6—N9	179.3 (3)	C14A—N7—C13A—C13B	4.8 (3)
C4—C5—C6—C1	−1.6 (4)	C14A—C8M—C15A—N8	4.2 (4)
C4A—N2—C3A—C2M	177.5 (3)	C14A—C8M—C15A—C15B	−176.8 (3)
C4A—N2—C3A—C3B	−0.9 (3)	C14A—C8M—C67—C68	−115.6 (3)
C4A—C3M—C5A—N3	−2.7 (4)	C14A—C8M—C67—C72	66.3 (4)
C4A—C3M—C5A—C5B	−179.2 (3)	C15A—N8—C16A—C5M	−176.9 (3)
C4A—C3M—C19—C20	−68.9 (3)	C15A—N8—C16A—C16B	1.6 (3)
C4A—C3M—C19—C24	111.1 (3)	C15A—C8M—C14A—N7	6.6 (5)
C4M—C6A—C6B—C5B	178.7 (3)	C15A—C8M—C14A—C14B	−178.6 (3)
C4M—C7A—C7B—C8B	177.5 (3)	C15A—C8M—C67—C68	68.2 (4)
C4M—C31—C32—C33	179.4 (2)	C15A—C8M—C67—C72	−110.0 (3)
C4M—C31—C36—C35	−179.9 (3)	C15A—C15B—C16B—C16A	0.7 (3)
C5A—N3—C6A—C4M	179.2 (2)	C16A—N8—C15A—C8M	178.0 (3)
C5A—N3—C6A—C6B	−1.3 (3)	C16A—N8—C15A—C15B	−1.1 (3)
C5A—C3M—C4A—N2	−5.5 (5)	C16A—C5M—C9A—N5	−11.1 (4)
C5A—C3M—C4A—C4B	172.6 (3)	C16A—C5M—C9A—C9B	171.7 (3)
C5A—C3M—C19—C20	113.7 (3)	C16A—C5M—C37—C38	−61.9 (4)
C5A—C3M—C19—C24	−66.4 (3)	C16A—C5M—C37—C42	119.2 (3)
C5A—C5B—C6B—C6A	2.5 (3)	C37—C5M—C9A—N5	170.0 (2)
C6—N9—C7—O1	4.5 (5)	C37—C5M—C9A—C9B	−7.2 (4)
C6—N9—C7—C8	−173.7 (3)	C37—C5M—C16A—N8	174.7 (2)
C6—C1—C1M—C1A	−68.3 (3)	C37—C5M—C16A—C16B	−3.5 (4)
C6—C1—C1M—C8A	108.6 (3)	C37—C38—C39—C40	0.2 (5)
C6—C1—C2—C3	1.6 (4)	C38—C37—C42—N13	−177.8 (3)
C6A—N3—C5A—C3M	−174.0 (2)	C38—C37—C42—C41	−1.4 (4)
C6A—N3—C5A—C5B	2.9 (3)	C38—C39—C40—C41	−0.6 (5)
C6A—C4M—C7A—N4	2.4 (4)	C39—C40—C41—C42	0.0 (5)
C6A—C4M—C7A—C7B	−175.4 (3)	C40—C41—C42—N13	177.6 (3)
C6A—C4M—C31—C32	−116.0 (3)	C40—C41—C42—C37	1.0 (4)
C6A—C4M—C31—C36	64.2 (3)	C42—N13—C43—O3	−6.5 (4)
C7—N9—C6—C1	160.0 (3)	C42—N13—C43—C44	172.6 (2)
C7—N9—C6—C5	−20.8 (4)	C42—C37—C38—C39	0.8 (5)
C7—C8—C9—C10	−178.6 (3)	C43—N13—C42—C37	−59.2 (4)
C7—C8—C12—N10	176.4 (3)	C43—N13—C42—C41	124.3 (3)
C7A—N4—C8A—C1M	177.7 (2)	C43—C44—C45—C46	−180.0 (3)
C7A—N4—C8A—C8B	−0.5 (3)	C43—C44—C48—N14	−179.7 (3)
C7A—C4M—C6A—N3	5.5 (4)	C44—C45—C46—C47	−0.5 (5)
C7A—C4M—C6A—C6B	−174.0 (3)	C45—C44—C48—N14	−1.2 (5)
C7A—C4M—C31—C32	62.6 (3)	C45—C46—C47—N14	−1.2 (6)
C7A—C4M—C31—C36	−117.2 (3)	C47—N14—C48—C44	−0.4 (5)
C7A—C7B—C8B—C8A	0.3 (3)	C48—N14—C47—C46	1.6 (5)
C8—C9—C10—C11	1.6 (6)	C48—C44—C45—C46	1.6 (5)
C8A—N4—C7A—C4M	−177.5 (2)	C49—C6M—C10A—N5	−171.3 (3)
C8A—N4—C7A—C7B	0.7 (3)	C49—C6M—C10A—C10B	6.4 (4)
C9—C8—C12—N10	−0.7 (5)	C49—C6M—C11A—N6	−171.5 (3)
C9—C10—C11—N10	0.0 (6)	C49—C6M—C11A—C11B	9.1 (4)
C11—N10—C12—C8	2.2 (5)	C49—C50—C51—C52	−0.3 (5)
C12—N10—C11—C10	−1.8 (5)	C50—C49—C54—C53	−0.7 (4)
C12—C8—C9—C10	−1.2 (5)	C50—C51—C52—C53	−0.6 (5)
C13—C2M—C3A—N2	179.2 (2)	C51—C52—C53—C54	0.9 (5)
C13—C2M—C3A—C3B	−2.7 (4)	C52—C53—C54—C49	−0.2 (5)
C13—C14—C15—C16	1.8 (5)	C54—C49—C50—C51	1.0 (5)
C14—C13—C18—C17	−1.7 (4)	C55—C7M—C12A—N6	176.0 (3)
C14—C15—C16—C17	−2.2 (5)	C55—C7M—C12A—C12B	−3.8 (4)
C15—C16—C17—C18	0.6 (5)	C55—C7M—C13A—N7	169.4 (3)
C16—C17—C18—C13	1.4 (4)	C55—C7M—C13A—C13B	−5.6 (4)
C18—C13—C14—C15	0.1 (4)	C55—C56—C57—C58	−0.1 (4)
C19—C3M—C4A—N2	177.3 (2)	C56—C55—C60—N15	175.3 (3)
C19—C3M—C4A—C4B	−4.5 (4)	C56—C55—C60—C59	−2.5 (4)
C19—C3M—C5A—N3	174.5 (2)	C56—C57—C58—C59	−1.1 (4)
C19—C3M—C5A—C5B	−2.0 (4)	C57—C58—C59—C60	0.6 (4)
C19—C20—C21—C22	−0.1 (4)	C58—C59—C60—N15	−176.5 (3)
C20—C19—C24—N11	176.7 (2)	C58—C59—C60—C55	1.3 (4)
C20—C19—C24—C23	−2.6 (4)	C60—N15—C61—O4	−3.1 (5)
C20—C21—C22—C23	−0.4 (4)	C60—N15—C61—C62	176.7 (3)
C21—C22—C23—C24	−0.7 (4)	C60—C55—C56—C57	1.9 (4)
C22—C23—C24—N11	−177.0 (2)	C61—N15—C60—C55	149.5 (3)
C22—C23—C24—C19	2.2 (4)	C61—N15—C60—C59	−32.8 (4)
C24—N11—C25—O2	3.6 (4)	C61—C62—C63—C64	−178.7 (3)
C24—N11—C25—C26	−176.7 (2)	C61—C62—C66—N16	−178.6 (3)
C24—C19—C20—C21	1.5 (4)	C62—C63—C64—C65	−2.5 (5)
C25—N11—C24—C19	136.9 (3)	C63—C62—C66—N16	1.5 (5)
C25—N11—C24—C23	−43.9 (4)	C63—C64—C65—N16	1.3 (5)
C25—C26—C27—C28	179.2 (3)	C65—N16—C66—C62	−2.7 (4)
C25—C26—C30—N12	−178.8 (3)	C66—N16—C65—C64	1.2 (5)
C26—C27—C28—C29	−0.4 (5)	C66—C62—C63—C64	1.1 (5)
C27—C26—C30—N12	−1.9 (4)	C67—C8M—C14A—N7	−169.3 (3)
C27—C28—C29—N12	−2.6 (5)	C67—C8M—C14A—C14B	5.5 (4)
C29—N12—C30—C26	−0.9 (4)	C67—C8M—C15A—N8	−179.8 (2)
C30—N12—C29—C28	3.2 (4)	C67—C8M—C15A—C15B	−0.9 (4)
C30—C26—C27—C28	2.5 (5)	C67—C68—C69—C70	−0.8 (6)
C31—C4M—C6A—N3	−176.0 (2)	C68—C67—C72—C71	−0.7 (5)
C31—C4M—C6A—C6B	4.5 (4)	C68—C69—C70—C71	0.0 (6)
C31—C4M—C7A—N4	−176.1 (2)	C69—C70—C71—C72	0.5 (5)
C31—C4M—C7A—C7B	6.1 (4)	C70—C71—C72—C67	−0.1 (5)
C31—C32—C33—C34	1.3 (4)	C72—C67—C68—C69	1.1 (5)
C32—C31—C36—C35	0.3 (4)	C1S—C2S—C3S—C4S	−62.6 (4)
C32—C33—C34—C35	−1.4 (4)	C2S—C3S—C4S—C5S	−174.5 (3)
C33—C34—C35—C36	0.9 (4)	C3S—C4S—C5S—C6S	178.0 (3)
C34—C35—C36—C31	−0.4 (4)	C73—C74—C75—C76	172.5 (10)
C36—C31—C32—C33	−0.8 (4)	C73A—C75A—C76A—C77A	−147 (3)
O3—C43—C44—C45	−48.0 (4)	C74—C75—C76—C77	−179.2 (8)
O3—C43—C44—C48	130.4 (3)	C74A—C73A—C75A—C76A	66 (3)
O4—C61—C62—C63	−52.4 (4)	C75—C76—C77—C78	179.8 (9)
O4—C61—C62—C66	127.8 (3)	C75A—C76A—C77A—C78A	8 (5)
N5—C9A—C9B—C10B	−2.1 (3)		

### Hydrogen-bond geometry (Å, º)

**Table:**

*D*—H···*A*	*D*—H	H···*A*	*D*···*A*	*D*—H···*A*
N13—H13···N10	0.88	2.16	3.015 (3)	163
N15—H15*A*···N12	0.88	2.14	2.963 (3)	156
N11—H11···O3	0.88	2.19	3.073 (3)	176
C27—H27···O3	0.95	2.50	3.140 (4)	125
N2—H2···N1	0.88	2.29	2.854 (3)	122
N4—H4···N3	0.88	2.31	2.869 (3)	121
